# Comprehensive Review on Thermoelectric Electrodeposits: Enhancing Thermoelectric Performance Through Nanoengineering

**DOI:** 10.3389/fchem.2021.762896

**Published:** 2021-12-21

**Authors:** Tingjun Wu, Jiwon Kim, Jae-Hong Lim, Min-Seok Kim, Nosang V. Myung

**Affiliations:** ^1^ Shanghai Institute of Microsystem and Information Technology, Chinese Academy of Sciences, Shanghai, China; ^2^ Materials Science and Chemical Engineering Center, Institute for Advanced Engineering, Yongin-si, Korea; ^3^ Department of Materials Science and Engineering, Gachon University, Seongnam-si, Korea; ^4^ Department of Chemical and Biomolecular Engineering, University of Notre Dame, Notre Dame, IN, United States

**Keywords:** electrodeposition, electroplating, thermoelectrics, nanoengineering, defect engineering

## Abstract

Thermoelectric devices based power generation and cooling systemsystem have lot of advantages over conventional refrigerator and power generators, becausebecause of solid-state devicesdevices, compact size, good scalability, nono-emissions and low maintenance requirement with long operating lifetime. However, the applications of thermoelectric devices have been limited owingowing to their low energy conversion efficiency. It has drawn tremendous attention in the field of thermoelectric materials and devices in the 21st century because of the need of sustainable energy harvesting technology and the ability to develop higher performance thermoelectric materials through nanoscale science and defect engineering. Among various fabrication methods, electrodeposition is one of the most promising synthesis methods to fabricate devices because of its ability to control morphology, composition, crystallinity, and crystal structure of materials through controlling electrodeposition parameters. Additionally, it is an additive manufacturing technique with minimum waste materials that operates at near room temperature. Furthermore, its growth rate is significantly higher (*i.e.,* a few hundred microns per hour) than the vacuum processes, which allows device fabrication in cost effective matter. In this paper, the latest development of various electrodeposited thermoelectric materials (*i.e.,* Te, PbTe, Bi_2_Te_3_ and their derivatives, BiSe, BiS, Sb_2_Te_3_) in different forms including thin films, nanowires, and nanocomposites were comprehensively reviewed. Additionally, their thermoelectric properties are correlated to the composition, morphology, and crystal structure.

## Overview of Thermoelectrics

**FIGURE 1:**
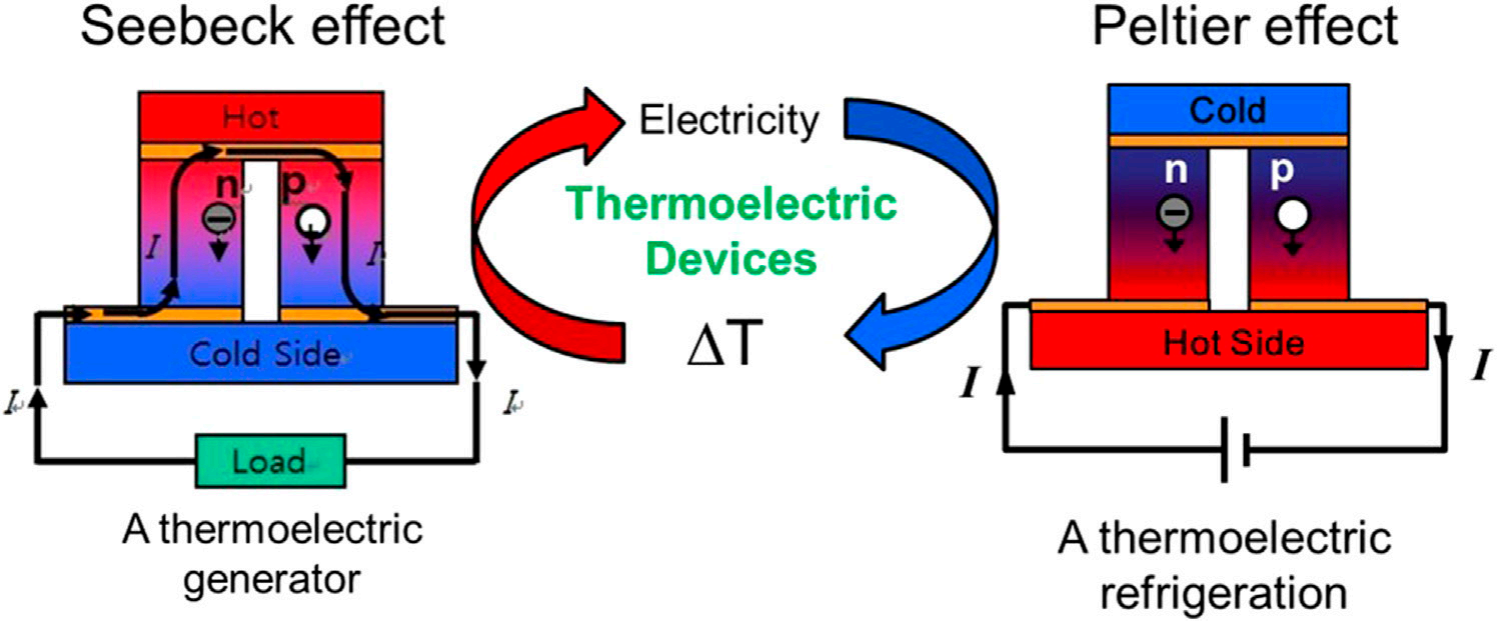
Schematic illustration of thermoelectric effect including the Seebeck effect and Peltier effect.

Thermoelectric power generators and coolers are based on the Seebeck and the Peltier effect, respectively, where the Seebeck effect allows direct conversion of temperature gradient into electricity (Figure 1). When establishing temperature gradient at the two sides of materials, charge carriers (*i.e.,* electronsin *n*-type semiconductor and holes in p-type semiconductor) would transfertransfer from hot side to cold side, which would create a voltage. voltage. The generated voltage, ΔV, is given by ΔV = S·ΔT, where S is the Seebeck coefficient and ΔT is the temperature difference. On the other hand, the Peltier effect is the generation of temperature gradient by applying electric energy. When electric energy is applied to the materials, charge carriers flow to one end of the thermoelectric materials. The charge carriers also transport energy, resulting in a temperature difference between the two ends.

In thermoelectric devices, the performance can be characterized by the dimensionless thermoelectric figure-of-merit (ZT), which is defined asas following equation:

$$ZT=\frac{S^{2}\sigma }{\kappa }T$$ (1)

where S is the Seebeck coefficient (V/K), *σ* is the electrical conductivity (S/m), κ is the thermal conductivity (W/mK) and T is the absolute temperature (K). (Zebarjadi et al., 2012) S^2^σ is defineddefined as the thermoelectric power factor (P. F.).

Additionally, the maximum energy conversion efficiency (*η*) of a thermoelectric device is defined as the energy produced toproduced the workwork (W) divided by the thermal energy consumed at the hot junction (Q), which is dependent on dependent onZT as well as the temperature difference of the hot and cold side (T_H_, T_C_). (Nolas et al., 2001; Snyder and Ursell, 2003; Sootsman et al., 2009; Zebarjadi et al., 2012).

$$\eta =\frac{W}{Q}=\frac{T_{H}-T_{C}}{T_{H}}\frac{\sqrt{1+ZT}-1}{\sqrt{1+ZT}+\frac{T_{H}}{T_{C}}}$$ (2)

Based on the definitions, high energy efficiency would be achieved by improving the thermoelectric power factor (S^2^σ) and suppressing the thermal conductivity. However, Seebeck coefficient, electrical conductivity and thermal conductivity are interdependent to each other, lead to significant difficulties to enhancing the energy efficiency (Szczech et al., 2011). For example, the Seebeck coefficient (S) is a function of the charge carrier (i.e., electrons or holes) effective mass and charge carrier concentration as shown in Eq. 3,

$$S=\frac{8\pi^{2}k_{B}^{2}}{3eh^{2}}m^{\text{*}}T{\left(\frac{\pi }{3n}\right)}^{\frac{2}{3}}$$ (3)

where e is the elementary carrier charge, k_B_ is Boltzmann constant, m* is the charge carrier effective mass, h is Planck’s constant, and *n* is the charge carrier concentration. The electrical conductivity (*σ*) is proportional to the product of carrier concentration and carrier mobility represented (Eq. 4).

$$\sigma =e\left(n_{e}\mu_{e}+n_{h}\mu_{h}\right)$$ (4)

where *e* is the elementary charge; *n*_*e*_ and *n*_*h*_ are the carrier concentrations of electrons and holes, respectively; *μ*_*e*_ and *μ*_*h*_ are the carrier mobility of electrons and holes, respectively. Based on these two equations, increasing the carrier concentration enhances the electrical conductivity, but decreases the Seebeck coefficient.

**FIGURE 2:**
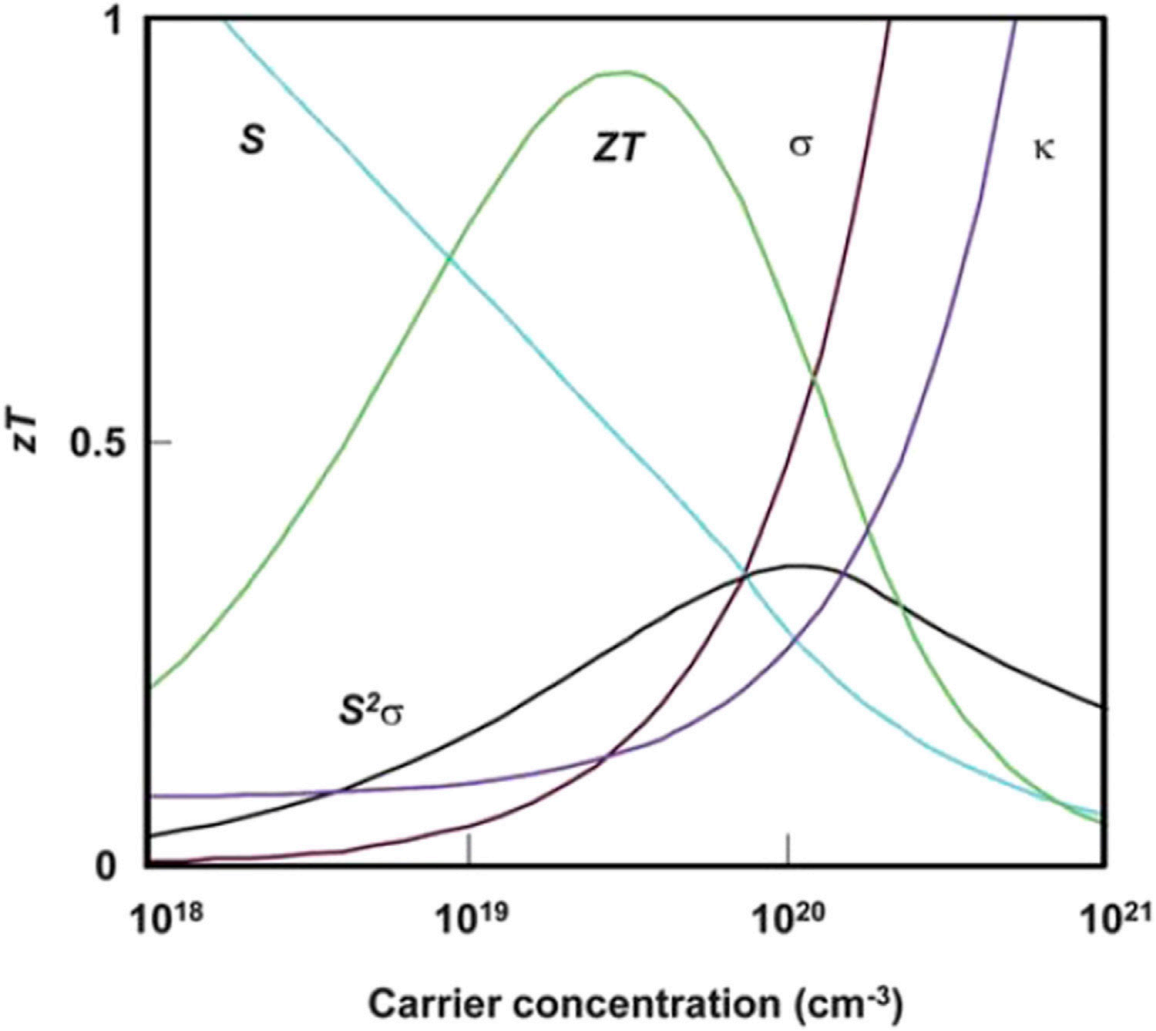
Interdependence of the Seebeck coefficient (S), electrical conductivity (*σ*), and thermal conductivity (*κ*) (Snyder and Toberer, 2008; Szczech et al., 2011).

The electrical conductivity (*σ*) and thermal conductivity (*k*) are also interdependent since thermal conductivity (*κ*) is combination of the lattice thermal conductivity (*κ*_l_) and electrical thermal conductivity (*κ*_e_). *κ*_e_ is proportional to the electrical conductivity $\;\left(\kappa_{e}=\sigma LT\right),\;\;$ by Wiedemann-Franz law (Szczech et al., 2011). Thus, increasing the carrier concentration increases both electrical conductivity and thermal conductivity. Figure 2 shows the interdependency of the Seebeck coefficient, the electrical conductivity and the thermal conductivity (Snyder and Toberer, 2008; Szczech et al., 2011).

**FIGURE 3:**
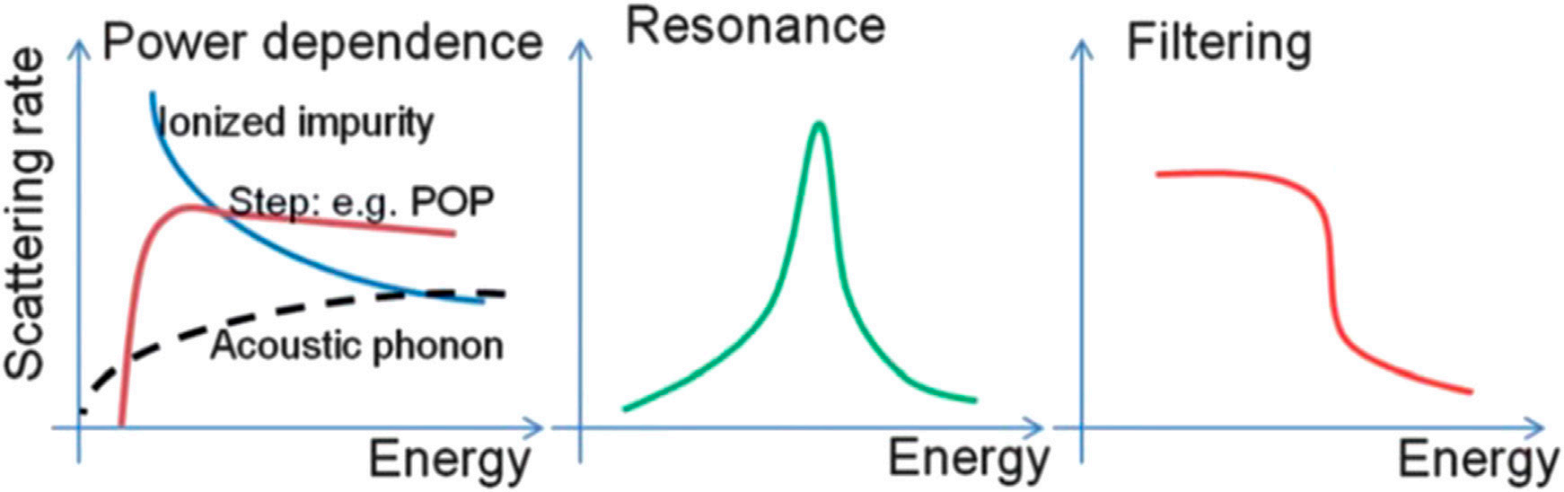
Several possible behaviors of total relation rate (1/τ(E)) in a few k_B_T window. (Zebarjadi et al., 2012).

In order to overcome this intrinsic demerit, numerous researchers endeavored to independently control these parameters by utilizing quantum confinement effect, phonon scattering effect, and energy filtering effect. Historical approaches to enhance the ZT have been focused on altering phonon scattering mechanism, called phonon-glass electron-crystal (PGEC), by introducing complex lattice structures such as skutterudites, superlattices, heterostructure, and nanocomposites. The enhancement of ZT in these systems was mainly achieved by reducing the thermal conductivity due to the increased phonon scattering at the interfaces. (Poudel et al., 2008). However, there is a limit for reducing the lattice thermal conductivity. Recent advancements have been achieved by incorporating metallic and/or semiconducting nanoparticles in thermoelectric matrices. (Hsu et al., 2004; Zeng et al., 2007; Zide et al., 2006). The distortions of the density of states (DOS) near Fermi level as results of carrier localization, resonant state, and carrier filtering effect fulfilled the sharp increase of the Seebeck coefficient without suppressing electrical conductivity. As shown in Figure 3, the large Seebeck coefficient can be dependent on the behavior of the scattering rates (1/τ) as a function of energy in the materials (Zebarjadi et al., 2012). The 1/τ, which is inverse function of the energy dependence of the relaxation times (*τ* = *τ*_0_Er), where the exponent *r* is called the scattering parameter. This scattering parameter, which is determined by different scatterings for example, in the case of acoustic phonon scattering, the *r* is -1/2, and weak impurity scattering, the *r* is 3/2. Therefore, an increase of the scattering parameter leads to an increase in the slope of the differential conductivity, thus also in the Seebeck coefficient.

Recently, the energy filtering effect where the creation of band bending induced by charge transfer at the interfaces causes the energy-dependent scattering of charge carriers was used to decouple S and σ . In the concrete, a barrier height (*E*_*b*_) can be generated on the pathways of charge carriers by interfaces, where the charge carriers with higher energy would pass though but the charge carriers with low energy would be scattered. The carrier charge scattering, which is dependent on energy, would improve Seebeck coefficient, owing to its correlation with the energy derivative of the relaxation time at the Fermi energy;

$$\text{S}=\;\frac{\pi^{2}kB^{2}T}{3e}\left(\frac{\partial lnN\left(E\right)}{\partial E}+\frac{\partial ln\tau \left(E\right)v{\left(E\right)}^{2}}{\partial E}\right)E_{F}$$ (5)

$$\tau^{-1}\left(E\right)=\;\frac{V_{b}^{2}x}{R}E^{-3/2}=\frac{e\mu }{m^{*}}$$ (6)

Where *υ(E)* is the velocity of average charge, *N(E)* is the density of states, *τ(E)* is the charge carrier relaxation time. Furthermore, as shown in Eq. 6, the carrier relaxation time is proportional to the barrier potential (*V*_*b*_) by inversion, which means tailoring a potential barrier to an effective height can be utilized to enhancing the Seebeck coefficient. (Faleev and Léonard, 2008; Martin et al., 2009; Ko et al., 2011; Sumithra et al., 2011; Narducci et al., 2012; Zhang et al., 2012).

## Electrodeposition of Thermoelectric Materials

Xiao et al. (2008) and Boulanger (2010) reviewed the advances in the electrodeposition of thermoelectric materials in 2008 and 2010, respectively, where major focus was devoted to electrochemistry of thermoelectric materials. Rostek et al. (2015) and others (Snyder et al., 2003; Wang et al., 2013; Roth et al., 2014; Shin and Oh, 2015; Uda et al., 2015; Pelz et al., 2016) reviewed the advancement of electrodeposition of Bi_2_(Te,Se)_3_ and (Bi,Sb)_2_Te_3_ thin films and electrodeposition-based processes to form TE microdevices.

Here, the latest development of various electrodeposited thermoelectric thin films and nanostructured materials (*i.e.,* Te, PbTe, Bi_2_Te_3_, BiSe, BiS, Sb_2_Te_3_, Cu_2_Se, CoSb_3_, Ag_8_SnS_6_, and their derivatives) were comprehensively reviewed in last 10 years. Especially, their thermoelectric properties were summarized and correlated to their composition, morphology, and crystal structure (Table 1).

**TABLE 1:** Correlation of material composition and microstructure with electrical and thermoelectric properties.

Ref	Materials	Morphology	Microstructure (Crystalline\diameter)	Preferred orientation	Grain size (nm)	Seebeck coefficient (µV K^−1^)	Electrical conductivity (S cm^−1^)	Thermal conductivity (W m^−1^ K^−1^)	Power factor (µW K^−2^ m^−1^)	ZT	Measure-temp.(K)
Abad et al. (2015)	Te	Thin film	Poly-crystalline		27 ± 3	285	12.5	1	280	0.09	RT
		Thin film	Poly-crystalline		43 ± 4	285	43.7	1	82	0.03	RT
Jiang et al. (2012)	Te	Thin film		(003)		342					473
Wu et al. (2016a)	Pb_49_Te_51_	Thick film		(220)		524	0.14		3.9		296
Lee et al. (2008)	Bi_2_Te_3_	Nanowires		(110)		30					RT
Li and Wang, (2009)	Bi_0.22_Sb_1.48_Te_3.30_	Thin film				119	78.7		111.5		RT
Diliberto et al. (2008)	Bi_1.93_Te_3.07_	Film		(110)		−65	833.3		352		RT
Suresh et al. (2009)	Bi_2_Te_3_	Thin film		(111)	83	−28.1					313
Kim and Oh, (2009)	Bi_2_Te_3_	Film		(110)		−51.6			710		RT
	Sb_2_Te_3_	Film				52.1			170		RT
Mannam et al. (2009)	n-Bi_2_Te_3_	Nanowires		(110)		−318.7					300
	p-Bi_2_Te_3_	Nanowires		(110)		117					300
Kim and Oh (2010a)	Bi_39_Te_61_	Film				−67	1,204.8		540		RT
	Sb_35_Te_65_	Film				63	179.5		70		RT
Lee et al. (2010)	Bi_2_Te_3_	Nanowires				53	1,690		476.3		RT
Richoux et al. (2010)	Bi_0.38_Sb_1.43_Te_3.19_	Film			40	230	54.3		287		RT
Li and Wang, (2010)	Bi_0.49_Sb_1.53_Te_2.98_	Film		(015)		185	299.4				RT
Chen et al. (2010)	Bi_2_Te_3_	Nanowires				−65		0.75		0.45	300
	Bi_2_Te_3_	Nanowires				−75		0.75		0.9	350
Li et al. (2010a)	Bi_0.5_Sb_1.5_Te_3_	Film				85					RT
Rostek et al. (2011)	Bi_39.6_Te_60.4_	Thin film				−55					RT
Ma et al. (2011)	Bi_39.3_Te_60.7_	Thin film				−58.3	1,036		352.2		RT
Pinisetty et al. (2011a)	Te-rich Bi_2_Te_3_	Nanowire				−48 ± 2.3					RT
	Te-rich Bi_2_Te_3_	Nanotube				−63 ± 1.9					RT
Zhu and Wang, (2012)	Bi_0.40_Sb_1.28_Te_3.14_Se_0.18_	Thin film				158	138.9				RT
Zou et al. (2012)	Bi_2_Te_2.7_Se_0.5_	Thin film				−92	95.0		80.4		RT
Kim and Oh (2013)	Bi_39.5_Te_60.5_	Thick film				−59.8	1,408.5		506		RT
	Sb_42.9_Te_57.1_	Thick film				485.4	210.5		4,960		RT
Manzano et al. (2013)	Bi_46_Te_54_	Film		(110)		−72	851.1		440		380
Rashid et al. (2013)	Bi_2_Te_3_	Film		(110)	43.1	−169.49			1737		RT
	Bi_2_Te_3_			(110)	21.1	112.3			443		RT
Cao et al. (2013)	Bi_2_Te_3_	Thin film				−140	600		1,247		RT
Wu et al. (2013)	Bi_2_Te_3_	Film				−120					RT
Yoo et al. (2013a)	Bi_11_Te_10_	Thin film		(015)		−70			336.2		RT
Wang et al. (2013)	Bi_0.47_Sb_1.44_Te_3.09_	Film				145			220		RT
	Bi_1.98_Te_2.73_Se_0.29_	Film				−83.2			210		RT
Rashid and Chung, (2013)	Bi_1.9_Te_3.1_	Thin film		(110)	28	−61.215			820		RT
Zou et al. (2014)	Bi_2_Te_2.65_Se_0.44_	Film		(110)		−88	142		110.0		RT
Maas et al. (2014)	Bi_2_Te_3_	Thick film				−90	512.8		500		RT
Szymczak et al. (2014)	Bi_2_Te_3_	Film		(110)		−70	75.2				RT
Jiang et al. (2014)	Bi_2_Te_3_/PEDOT:PSS/Bi_2_Te_3_	Film				16	402.5	0.17		0.017	RT
Caballero-Calero et al. (2014)	Bi_37.7_Te_62.3_	Film		(110)		−80					358
Matsuoka et al. (2015)	Bi_54_Te_46_/BiSe	Layered structure			38/15	−46			144		RT
Caballero-Calero et al. (2015)	Bi_1.7_Te_3.1_Se_0.2_	Thin film		(110)		−100					353
Zhou et al. (2015)	Bi_2_Te_3_	Thin film		(110)		−81	520		340	0.16	RT
Li et al. (2015)	Bi_0.5_Sb_1.5_Te_3_	Nanowires	Dia. 67nm	(110)		143	480	0.28		1.14	330
Uda et al. (2015)	Bi_37.5_Te_62.5_	Film				−81.9	526.3		354		RT
Chang et al. (2015)	Bi_39_Te_61_	Nanowires	Dia. 60nm			71	390		195.8		300
Shin and Oh (2015)	Bi_39.4_Te_60.6_	Film				−59.5	1,587.3		559		RT
	Sb_43.1_Te_56.9_	Film				441.2	281.7		5,480		RT
Kulsi et al. (2015)	Bi_1.6_Te_3.4_	Thin film		(018)	55	−29	4,033		340	0.28	RT
Lei et al. (2016a)	Bi_2_Te_3_	Thick film		(110)		−200	400		1,600		RT
Yang et al. (2016)	Te-Bi-Sb	Film				32.9			34		RT
Na et al. (2016)	Bi_2.17_Te_2.83_	Film		(110)	35.7	−146	691		1,473		RT
Kulsi et al. (2016)	Bi-Te	Film			127	−32	1,247	0.46	130	0.08	RT
Lei et al. (2016b)	Bi_2_Te_3_	Thick film		(110)	17	−80	330				RT
Manzano et al. (2016)	Bi_2_Te_3_	Film		(110)		−-58	670		225	0.056	300
Jagadish et al. (2015)	Bi_2_Te_2.53_	Film				−20					RT
Lal et al. (2017)	(Sb_0.68_Bi_1.10_)_2_Te_3.25_	Film			17.6	11					RT
Kang et al. (2017)	Bi_2_Te_3_	Thick film				−72.3	1,408		732		RT
Lei et al. (2017)	Bi_0.5_Sb_1.5_Te_3_	Thick film		(015)	17	150	100		230		RT
Wu et al. (2017b)	Bi_2_Te_3_-silica particle	Film				78					RT
Xiaolong and Zhen, (2014)	Bi_2_Se_3_	Thick film				20	1,309		52.57		RT
Jagadish et al. (2016)	Bi_2_S_2.34_	Film				−16.3					RT
Kim and Oh, (2010b)	Sb_2_Te_3_	Thin film				322					RT
Pinisetty et al. (2011b)	Sb_2_Te_3_	Nanowires	Dia. 100nm		36	359					300
	Sb_2_Te_3_	Nanotubes	Dia. 400nm		43	332					300
Lim et al. (2011)	Sb_2_Te_3_	Film		(015)		118					RT
Qiu et al. (2011)	Sb_2_Te_5_	Thin film				532			1,580		RT
Schumacher et al. (2012)	Sb_39.08_Te_60.92_	Film		(015)	543	161	280		726		RT
Lim et al. (2012b)	Sb_5_Te_8_	Thin film		(015)		118			44.2		473
Yoo et al. (2013c)	Sb_2_Te_3_	Thin film				280			100		RT
Kim et al. (2016)	AgSbTe_2_	Thin film	Nano-crystalline			300			553		RT

### Electrodeposition of Tellurium

Electrodeposition of tellurium has been investigated in both acidic and alkaline media. Qiu et al. (1989) electrodeposited Te thin films with a thickness up to 4 µm on monocrystalline tellurium substrate from a TeO_2_-saturated aqueous solution. The thickness was relatively uniform. The needle-like surface morphology with random crystal orientation was observed when deposited on (10 $\bar{1}$ 0) surfaces. At high current densities, polycrystalline films consisting of 1 µm blades with random crystal orientation were produced (Qiu and Shih, 1989).

Suggs et al. (1991) investigate the electrochemical nucleation and growth of Te on gold (Au) (100) surface in acidic sulfate baths (*i.e.*, 0.4 mM TeO_3_^2-^ in X M H_2_SO_4_). Under potentiodynamic deposition, Te initially electrodeposited under underpotential deposition (UPD). As the deposition potential becomes more cathodic, Te electrodeposits under overpotential deposition (OPD) to from three dimensional nuclei. (Suggs and Stickney, 1991).

Ikemiya et al. (1996) electrodeposited Te films on Au (100) and Au (111) from acidic sulfate solutions (0.1 mM HTeO_2_^+^ + 0.05 M H_2_SO_4_). The atomic structures and growth morphologies of the films were investigated by *in situ* atomic force microscopy. Accordingly, the atomic structure of the Te deposits was independent to the substrate crystal orientation, this support the conclusion that the surface diffusion process of Te adsorbed atoms is rate-limiting steps (Ikemiya et al., 1996).

Yagi et al. (1996) electrodeposited Te in acidic perchlorate solutions with 0.1 M HClO_4_ and 0.5 mM TeO_2_ using polycrystalline gold as substrate. AuAdditionally, *in situ* optical second harmonic (SH) generation at two different excitation wavelengths was utilized. On 1,064 nm excitation, the SH signal varied with the surface coverage of Te (Yagi et al., 1996).

Sorenson et al. synthesized tellurium atomic layers on Au (110) by electrodeposition in the acidic bath (*i.e.,* 0.25 mM TeO_2_ + 20 mM H_2_SO_4_). Additionally, the phase transitions associated with those layers was investigated. The voltammetry indicates two sub-monolayer deposition features and one for bulk. The result of the slow deposition kinetics is that surfaces composed of a single atomic layer structure are not observed. (Sorenson et al., 1999; Sorenson et al., 2001)

Jiang et al. electrodeposited Te film on polyaniline-coated macroporous phenolic foam in the solution with 1 M HNO_3_ and 10 mM HTeO_2_^+^. The deposited film was composed of columnar structures and had a growth direction along c-axis direction (Jiang et al., 2011). The highest Seebeck coefficient achieved for the macroporous Te film is 342 μV/K at 473 K (Jiang et al., 2012).

Abad et al. (2015) electrodeposited Te films from acidic nitrate baths (*e.g.,* 10 mM HTeO_2_^+^ and 1 M HNO_3_) with sodium lignosulfonate (SLS) as additives. The presence of SLS reduced the average grain size resulted in higher electrical resistivity (∼798 µΩ m) compared to Te electrodeposits (∼229 µΩ m) in the absence of SLS. The Seebeck coefficient values were about 285 µV/K for both samples which resulted in the power factor of 280 µW/(mK^2^) and 82 µW/(mK^2^) without SLS and with SLS, respectively, at room temperature (Abad et al., 2015).

Ha et al. (2000) reported the electrochemical behavior of tellurium in alkaline baths (*e.g.,* 10 mM TeO_3_^2-^ in2.5 M NaOH). In this bath, Te was able to electrodeposit between -0.8 V and -0.95 V vs. Hg/HgO, but the Te morphology was porous with needle-like radial growth (Ha et al., 2000).

Sadeghi et al. (2008) electrodeposited Te using a nickel-coated copper as substrate in alkaline plating baths. The influence of current density, temperature, and pH were systematically studied. They found that the optimum conditions to electrodeposit Te was: 6 g/L (37.6 mM) TeO_2_, pH of 10, and DC current density of 8.55 mA/cm^2^ at room temperature (Sadeghi et al., 2008).

Our group also demonstrated the ability to electrodeposit thick Te films from alkaline baths (Wu et al., 2017a) where the applied potentials were optimized to electrochemically reduced TeO_3_^−2^ (*aq*) to Te_(s)_ without further reduction of Te to Te_2_^2-^ (*aq*). The XRD data revealed that the preferred orientation of thick Te films altered from (001) to (101) as the applied potential varied from −0.9 V to −1.0 V. The optimum pH ranges to deposit compact thick films was between 11.3 and 12.5. Additionally, sufficient magnetic agitation is also essential to deposit compact films. The average grain size ranged from 66 to 135 nm where larger grain size resulted in lower carrier concentration (*e.g.*, *n* = 7.1 × 10^18^ cm^−3^) which might be due to lower defect density. The Highest deposition rate (upto 130 µm/h) with high current efficiency (upto 85%) was achieved by adjusting deposition conditions. Additionally, galvanic displacement reaction which is another facile method to synthesize various nanostructured Te was investigated (Chang et al., 2010; Chen et al., 2010; Hangarter et al., 2010; Lee et al., 2011; Jung et al., 2012; Elazem et al., 2013; Park et al., 2013; Suh et al., 2014; Suh et al., 2017).

### Electrodeposition of Lead Telluride Based Materials

PbTe is also a narrow band-gap semiconductor with E_g_ of 0.31 eV measured at room temperature and a rock-salt crystal structure. PbTe can be n-type or p-type as a result of departures from stoichiometry (*n*-type for Pb-rich PbTe, while p-type for Te-rich PbTe). (Dughaish, 2002). The state-of-the-art commercially available PbTe based thermoelectric materials have the highest ZT of ∼0.8 at ∼ 600 K, which makes the materials a good candidate for thermoelectric application in the middle-high temperature range.

The Electrodeposition of PbTe was investigated by several groups. Saloniemi et al. reported electrodeposition of Te-rich PbTe thin films in alkaline electrolytes containing TeO_2_, disodium salt of ethylenediaminetetraacetic acid (EDTA), and Pb(CH_3_COO)_2_ethylenediaminetetraacetic. They utilized various electrochemical analysis methods including cyclic voltammetry and quartz crystal microbalance to investigate the electrodeposition of PbTe. They observed that Te-rich PbTe deposition through UPD of Pb on Te *via* six electron reduction (Saloniemi et al., 1998). The reduction of the PbEDTA^2-^ complex to Pb_(0)_ was a two-electron reaction whereas Te deposits *via* a four-electron reaction. As the potential becomes more negative, the film becomes powdery and Te_(0)_ further reduced to Te_2_^2-^ as the deposition potential becomes more negative (Saloniemi et al., 2000).

Miranda et al. electrodeposited polycrystalline PbTe thin films on porous silicon from alkaline solutions with EDTA as a complexing agent for Pb. They were able to deposit PbTe thin films with the average grain size of 100 nm (Miranda et al., 2004).

Qiu et al. (2005) synthesized uniform and single-crystalline PbTe nanorods with a diameter in the sub-10-nm regime at ambient conditions using sonoelectrochemical method. In the experiment, the Pb^2+^ and TeO_3_^2-^ ions concentration were fixed at 10 mM, and the solution pH was kept at approximately 8. Nitrilotriacetic acid (NTA) was used as a complex reagent. The composition of PbTe can be controlled by ratio of precusor ion/ligand concentration. When the [Pb^2+^]/[NTA] changed from 0.20:1 to 0.10:1 to 0.05:1, the composition of deposits changed from pure PbTe to a mixture of PbTe/Te to pure Te (Qiu et al., 2005).

Yang et al. (2008) electrodeposited PbTe nanowire arrays using template which is patterned by lithographic method. The cross-section of the synthesized PbTe nanowires is rectangular, and the width and height of the nanowires can be tuned from 60 to 400 nm and 20–100 nm, respectively. Polycrystalline PbTe with face centered cubic crystal structure and was produced by a cyclic electrodeposition-stripping method, which have grain size ranged from 10 to 20 nm. The nanowires have a length over 1 mm (Yang et al., 2008).

Erdogan et al. (2009) electrodeposited stoichiometric PbTe thin films on Au (111) substrates from alkaline baths containing EDTA, Pb^2+^, and TeO_3_^2-^ ions. They observed two dimensional nucleation and growth with the preferred orientation of (200) (Erdoğan et al., 2009).

Li et al. (2008a) electrodeposited symmetrical PbTe dendritic structures in the solution containing 10 mM Na_2_TeO_3_, 5 mM Pb(NO_3_)_2_ and 0.1 M tartaric acid. The formation of the PbTe dendritic structure is affected by the potential oscillation. The morphology of particle with dendritic structures were star-like or trigonal, and the size of the particles were varied from 100 to 500 nm. The deposited PbTe structures had a band gap energy of about 0.272 eV (Li et al., 2008a).

Additionally, many other groups reported the results of characterization of PbTe electrodeposits based on various experimental conditions which are summarized on the Table 1. (Banga et al., 2008; Diliberto et al., 2008; Jung et al., 2011; Ni et al., 2011; Frantz et al., 2015; Wu et al., 2016a; Frantz et al., 2016; Bae et al., 2017).

### Electrodeposition of Bismuth Telluride (Bi_2_Te_3_) Based Materials Including BiTe, BiSbTe, BiTeSe and BiSbTeSe

**FIGURE 4:**
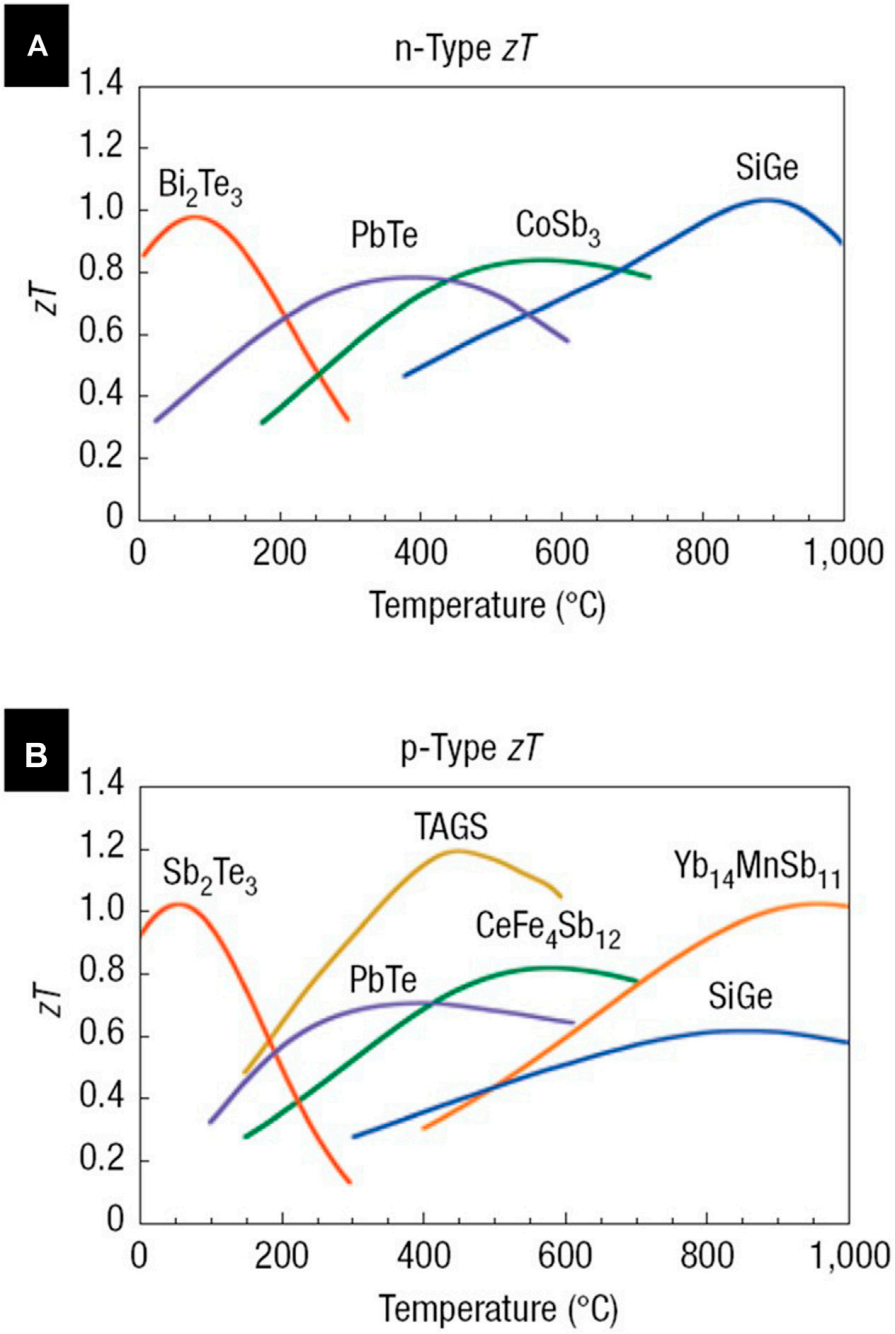
Thermoelectric performance (ZT) of the state-of-art commercial thermoelectric materials: **(A)** n-type and **(B)** p-type, as function of temperature (Snyder and Toberer, 2008).

Bi_2_Te_3_ with a bandgap of 0.16 eV is an excellent candidate for TE application near room temperature range (Figure 4). Electrodeposition of Bi_2_Te_3_ was investigated by various groups.

Wang et al. (2008) synthesized high-density thermoelectric Bi_2_Te_3_/Sb heterostructure nanowire arrays with diameter of tens using AAO template-directed pulsed electrodeposition. The electrolyte included 12 mM TeO_2_, 4 mM Bi(NO_3_)_3_, 0.1 M Sb_2_O_3_, 0.5 M K_2_C_6_H_5_O_7_, 1 M C_6_H_8_O_7_, and 2 M HNO_3_. Additionally, was used as template (Wang et al., 2008).

Li et al. synthesized the hierarchical Bi_2_Te_3_ nanostructures by electrodeposition in the solution with 10 mM Na_2_TeO_3_, 5 mM Bi(NO_3_)_3_, 10 mM tartaric acid and 1 M HNO_3_ at room temperature (Li et al., 2008b; Li et al., 2008c).

Liu and Li (2008) reported that electrodeposited Bi_2_Te_3_ films had a preferential orientation of (110) and platelet grain morphology. The grain morphology changed from single-to multi-order platelets, and the texture decreased when the deposition potential became more negative, which was explained by considering geometrical selection growth and (1ī010) (ī 105) twinning of Bi_2_Te_3_ crystals (Liu and Li, 2008).

Glatz et al. (2008) electrodeposited Bi_2+x_Te_3−x_ by combining potential controlled deposition pulses with galvanostatic-controlled resting pulses. The deposited had a uniform stoichiometry composition along the entire thickness. A deposition rates of 50 µm/h was achieved, and Layers thickness of 800 μm was obtained. The composition of Bi_2+x_Te_3−x_ can be controlled by varying Bi ion concentration in the electrolyte with 80 mM HTeO_2_^+^ and 2 M HNO_3_. Bath n-type and p-type Bi_2+x_Te_3−x_, which is determined by Seebeck coefficients, was deposited (Glatz et al., 2008).

Lee et al. (2008) electrodeposited Bi_2_Te_3_ nanowires arrays using AAO as template by potentiostatic, galvanostatic, and pulsed method in aqueous solution at room temperature. Uniform Bi_2_Te_3_ nanowire arrays with highly oriented crystalline structure was synthesized, The bandgap of the deposited can be controlled from 0.21 to 0.29 eV by different relaxation times in the pulsed electrodeposition. The electrical resistances increased slightly with increasing temperatures, which was owing to enhanced carrier-phonon scattering. All samples showed a positive Seebeck coefficient (12–33 µV/K). (Lee et al., 2008).

Li et al. electrodeposited Bi_x_Sb_2-x_Te_y_ in nitric acid and hydrochloric acid solutions. A composition of Bi_0.5_Sb_1.5_Te_3_ was gained in both acid solutions with significantly different morphology. The Bi_0.47_Sb_1.36_Te_3.17_ thin film prepared in the nitric acid solution has the highest Seebeck coefficient of 213 µV/K. The Bi_0.22_Sb_1.48_Te_3.30_ film prepared in the hydrochloric acid solution has the highest power factor of 111.5 µW/(mK^2^), which had an electrical resistivity of 1.27 × 10^–4^ Ω m and Seebeck coefficient of 119 µV/K (Li and Wang, 2009).

Diliberto et al. (2008) synthesized Bi_2_Te_3_ thin films using pulsed electrodeposition from electrolytes of 20 mM Te(IV) ion and 1 M HNO_3_. The Bi ion concentration was varied, where increasing Bi concentration in the electrolyte would lead to higher Bi composition. The results also indicated that pulsed electrodeposition would improve the morphology and the electrical conductivity of films compared to direct electrodeposition. The film near stoichiometry (Bi_1.93_Te_3.07_) have a Seebeck coefficient of -65 µV/K (Diliberto et al., 2008).

Zhu et al. (2008) synthesized Bi_2_Te_3_ thin sheets on Au by electrochemical atomic layer epitaxy method using Bi solution with 0.25 mM Bi(NO_3_)_3_ and 0.1 M HClO_4_, and Te solutions with 0.25 mM TeO_2_ and 0.1 M HClO_4_. The bandgap of the Bi_2_Te_3_ film was 0.33 eV measured by Fourier transform infrared spectroscopy. Compared to the bulk Bi_2_Te_3_ single crystal, the bandgap is blue shifted (Zhu et al., 2008).

Mavrokefalos et al. (2009) reported electrodeposition of n-type Bi_2_Te_3_ nanowires (NW). The results showed that monocrystalline NWs have higher electrical conductivity and thermal conductivity than polycrystalline NWs. Additionally, the carrier mobility of the monocrystalline NW is about 2.5 times higher than that of the polycrystalline NW, but it about 19% lower than that of bulk materials. The electron mean-free path was decreased from 61 nm for bulk materials to 40 nm for the 52 nm nanowires, which is owing to electron scattering specularity parameter by nanowire surface is 0.7. Furthermore, the thermal conductivity of the polycrystalline nanowires is lower. The ZT is about 0.1 at 400 K for both monocrystalline and polycrystalline NWs (Mavrokefalos et al., 2009).

Li et al. electrodeposited Bi_0.5_Sb_1.5_Te_3_ thin film from nitric acid baths. The results show that electrodeposition mechanism varied with applied potential, where at low applied potential, Te was deposited because of electrochemical reduction of HTeO_2_^+^, while at more negative applied potential the reduction reaction of Bi^3+^ with Te occurred with formation of Bi_2_Te_3_. Additionally, when the applied potential is negative enough, formation of Bi_0.5_Sb_1.5_Te_3_ compound took place (Köse et al., 2009).

Li et al. examined the electrodeposition of Bi_2_Te_3_ in a solution containing TeCl_4_, Bi(NO_3_)_3_ and dimethyl sulfoxide (DMSO) by combining cyclic voltammetry with electrochemical quartz crystal microbalance. The results indicated Te^4+^ concentrations in and applied potential had an effect on Bi_2_Te_3_ composition. Bi_2_Te_3_ was electrodeposited in applied potential between −0.2 and −0.8 V vs. Ag/AgCl with 10 mM Te^4+^ and 7.5 mM Bi^3+^. However, Te-rich Bi_2_Te_3_ were electrodeposited at applied potential between -0.2 and -0.8 V vs. Ag/AgCl in the solution with 50 mM Te^4+^ and 37.5 mM Bi^3+^ (Li, 2009).

Suresh et al. (2009) electrodeposited Bi_2_Te_3_ thin films at various pH values in HNO_3_ solution of Bi (NO_3_)_3_ and TeO_2_. The increase in pH resulted in a decrease in grain size and the film morphology transformed from dispersed nanoparticles to connected chain-like nanostructures as pH was increased. At the temperature between 300 and 425 K, the data showed a four-times increase in Seebeck coefficient between its maximum and minimum value as the solution pH changes from 1 to 3.5, which is attributed to the improved connectivity of the nanostructures at higher pH (Suresh et al., 2009).

Kim and Oh (2009) electrodeposited n-type Bi_2_Te_3_ and p-type Sb_2_Te_3_ films. The n-type Bi_2_Te_3_ had a power factor of 7.1 × 10^–4^ W/(K^2^·m) with a Seebeck coefficient of −51.6 μV/K, which was electrodeposited at applied potential of −0.05 V with 25 mM Bi ion and 25 mM Te ion. Additionally, The p-type Sb_2_Te_3_ film had a power factor of 1.7 × 10^–4^ W/(K^2^·m) with a Seebeck coefficient of 52.1 μV/K, which is deposited at applied potential of 0.02 V in the solution containing 63 mM Sb ion and 7 mM Te ion. (Kim and Oh, 2009).

Mannam et al. (2009) electrodeposited Bi_x_Te_y_ nanowires from aqueous acidic solutions containing different [Bi^3+^]/[HTeO_2_^+^] (20/20 and 20/10 mM) with 2.5 M HNO_3_. The nanowires deposited at low applied potentials had a dominant orientation of (110) according to the XRD pattern. In both electrolytes, n-type nanowires were deposited. However, p-type nanowires can be deposited only in the [Bi^3+^]/[HTeO_2_^+^] = 20/10 mM solution. Nanowires formed in the 20/10 mM electrolyte showed at transition from intrinsic to extrinsic. The Seebeck coefficient of -318.7 and 117 μV/K were achieved for n-type and p-type Bi_x_Te_y_ nanowires, respectively (Mannam et al., 2009).

Kuleshova et al. (2010) electrodeposited BiSbTe films in nitric acid baths. In the electrolyte, sodium ligninsulfonate was added as surfactant, which would improve uniformity of the films as well as the thermoelectric properties. Additionally, the surfactant would also affect the composition of films, where Bi_0.32_Sb_1.33_Te_3_ was deposited in the solution with surfactant and Bi_0.35_Sb_1.33_Te_3_ was deposited without surfactant in the solution with 10 mM HTeO_2_^+^, 1 mM Bi^3+^, 20 mM Sb^3+^, 1 M HNO_3_, 0.1 M H_3_Cit and 50 mM Na_3_Cit (Kuleshova et al., 2010).

Ma et al. (2010) electrodeposited Bi_2_Te_3_ on stainless steel, in which the reaction mechanism and the effect of deposition parameters on composition and morphology were investigated. The CV results showed that onset potential for Bi_2_Te_3_ is more positive than Bi and Te deposition. Furthermore, the Te reduction reaction is kinetically hindered with the presence of Bi ions. (Ma et al., 2010).

Kim and Oh (2010a) synthesized p-type Sb_x_Te_y_ and n-type Bi_x_Te_y_ films by electrodeposition. The Bi_x_Te_y_ film with a thickness of 5.3 µm was electrodeposited in 1 M HNO_3_ solution at -0.05 V, which contained 50 mM Bi and Te ion. Moreover, the Bi/(Bi + Te) mole ratio is 0.5. The Sb_x_Te_y_ film with a thickness of 5.2 µm was electrodeposited at 0.02 V in the electrolyte, where the total concentration of Sb and Te ion is 70 mM and Sb/(Sb + Te) mole ratio is 0.9. The Bi_x_Te_y_ and Sb_x_Te_y_ films have an electrical conductivity of -67 and 63 µV/K(Kim and Oh, 2010a).

Lee et al. (2010) electrodeposited Bi_2_Te_3_ nanowires in AAO templates. They claimed the electrical conductivity can be improved from 0.053 to 0.169 × 10^6^ S/m by tailoring the structural properties. Meanwhile, the Seebeck coefficient can be enhanced from 46.6 μV/K to 55 μV/K. As a result, a power factor of 476.3 μW/(K^2^·m) was achieved (Lee et al., 2010).

Richoux et al. (2010) synthesized p-type (Bi_1-x_Sb_x_)_2_Te_3_ thermoelectric compounds by pulsed electrodeposition in the electrolyte with 1 M HClO_4_ and 0.1 M tartaric acid. The deposited film had a Seebeck coefficient of 150 μV/K. Additionally, pulsed electrodeposition method can be used to reduce resistivity of the films, where 200 μΩ m was achieved by pulsed electrodeposition method, compared to 5,000 μΩ m by direct-current electrodeposition method (Richoux et al., 2010).

Li et al. electrodeposited Bi_x_Sb_2−x_Te_y_ film by potentiodynamic electrodeposition technique from mixed dimethyl sulfoxide solution containing Bi(NO_3_)_3_·5H_2_O, TeCl_4_ and SbCl_3_. Their results showed that electrodeposition of Bi_x_Sb_2−x_Te_y_ can be realized in a wide range of applied potential. However, the films deposited at applied potential of -0.2 to -0.4 V achieved the highest S of 185 μV/K and the lowest electrical resistivity of 3.34 × 10^–5^ Ω m after annealing. Additionally, the deposited nano-crystalline Bi_0.49_Sb_1.53_Te_2.98_ film had a preferred orientation of (015) (Li and Wang, 2010).

**FIGURE 5:**
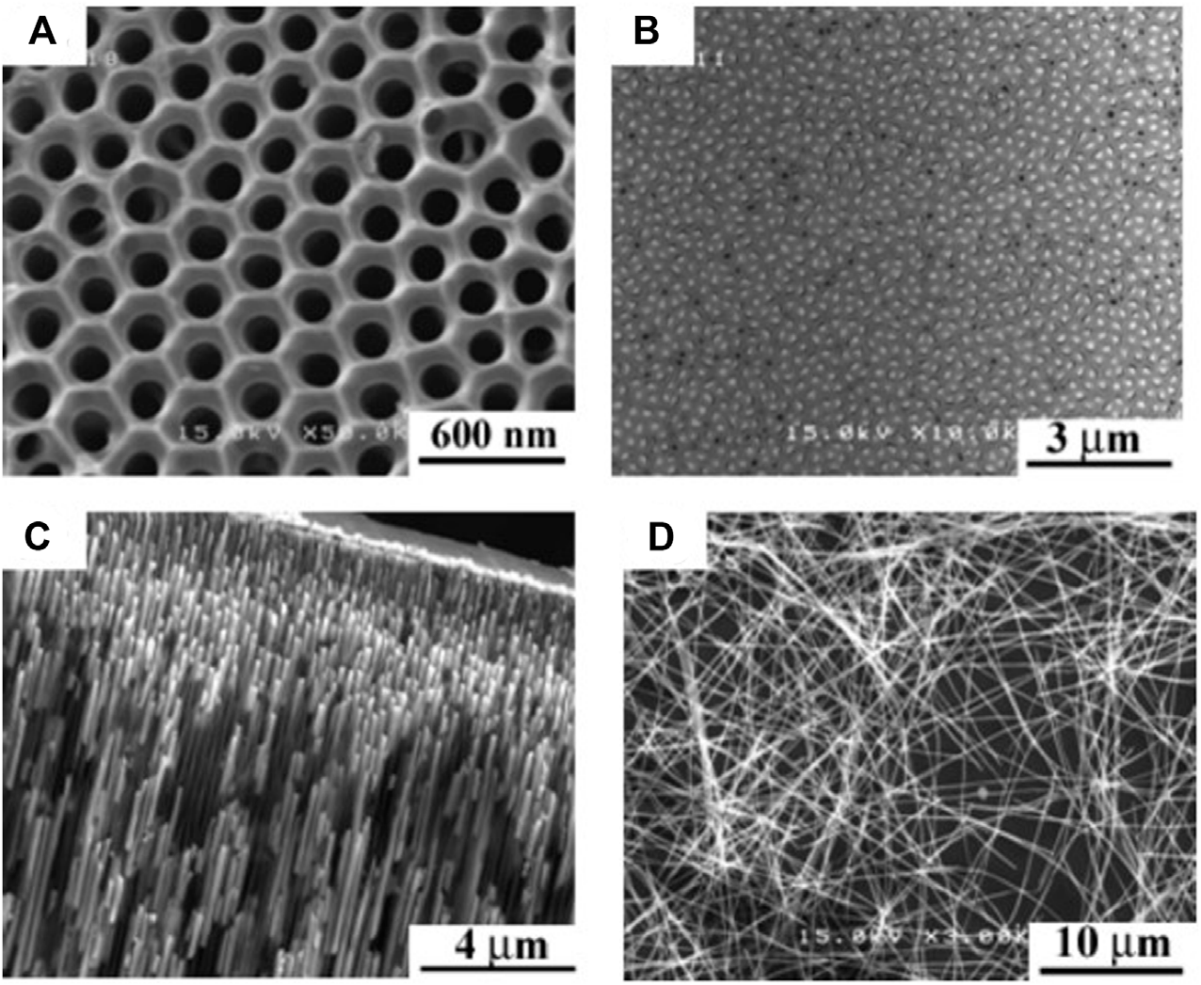
Scanning electron micrographs of AAO template and Bi_2_Te_3_ nanowires array: **(A)** AAO, **(B)** Top view of Bi_2_Te_3_ nanowires array, **(C)** Side view of Bi_2_Te_3_ nanowires array, **(D)** individual nanowires after dissolving AAO (Chen et al., 2010).

Chen et al. (2010) fabricated Te-rich n-type Bi_x_Te_y_ films and nanowires array with rhombohedral structure (Figure 5) by potentiostatically electrodeposition from nitric baths. The Seebeck coefficient was about -70 µV/K at 300 K and decreased monotonically with temperature. Additionally, thermal conductivity of 0.75 W/(mK) was obtained at 300 K. Aa a result, The ZT Bi_2_Te_3_ nanowire was 0.45 at 300 K and 0.9 at 350 K for (Chen et al., 2010).

Frantz et al. (2010) also synthesized polycrystalline Bi_2_Te_3_ nanowires with rhombohedral phase by electrodeposition using porous polycarbonate as template. Their results showed that dimethyl sulfoxide would help to increase the filling ratio to 80%. Moreover, DMSO in the electrolyte can help to improve the electrical conductivity of the nanowires (Frantz et al., 2010).

Gan et al. (2010) investigated Nanoscale Bi-Te particles with thermoelectric properties electrodeposited on copper substrate in 2.0 M HNO_3_. The atomic ratio 1:1 for Bi:Te in the alloy, which is equivalent to the weight percentage of Bi:Te = 62%:38% was confirmed from the EDS data (Gan et al., 2010).

Li et al. (2010a) investigated the electrochemical behavior Bi_x_Sb_2-x_Te_y_ in the solution consisting of 20 mM TeCl_4_, 20 mM Bi(NO_3_)_3_, 20 mM SbCl_3_, DMSO, and 0.1 mM KNO_3_. A smooth morphology was obtained for Bi_x_Sb_2-x_Te_y_ films deposited at different applied potential. The resistances reduced to about 0.04 Ω by post-annealing process. Seebeck coefficient of 85 μV/K was obtained for Bi_0.49_Sb_1.53_Te_2.98_ film (Li et al., 2010a).

Li et al. (2010b) electrodeposited Bi_2_Te_2.7_Se_0.3_ nanowire arrays using AAO as template in the electrolyte composing of 2 mM TeO_2_, 2.5 mM Bi(NO_3_)_3_, 0.3 mM SeO_2_ and 0.1 M HNO_3_. The post-annealing process was carried out at 300°C under an argon atmosphere. The single crystalline nanowires with diameter of about 14 nm were obtained (Li et al., 2010b).

Golgovici et al. (2010) synthesized BiSbTe films by electrodeposition in choline chloride (ChCl) and malonic acid based ionic liquids with a molar ratio of 1:1. The reaction temperature was controlled between 80 and 85°C. The concentration of Bi, Sb and Te ions ranged from 1.5 to 50 mM. The CV data showed that the Te reduction reaction happened first, followed by formation of binary or ternary compounds by codeposition. Furthermore, pulsed electrodeposition technique was also used to synthesize BiSbTe films (Golgovici et al., 2010).

Rostek et al. (2011) synthesized n-type Bi_2_Te_3_ films by electrochemical deposition. The films with composition near stoichiometric was deposited in the solution containing 20 mM Te ions and 30 mM Bi ions at a current density of 3.75 mA/cm^2^. The Seebeck coefficient of as-deposited Bi_2_Te_3_ films is about -55 μV/K. However, after annealed at 250°C for 60 h, the Seebeck coefficient increased to -130 μV/K(Rostek et al., 2011).

Ma et al. (2011) electrodeposited thin Bi_2_Te_3_ film onto stainless steel from acidic nitrate baths. The carrier concentration of the deposited films was ten times higher than the bulk Bi_2_Te_3_, while the Seebeck coefficient and Hall mobility is lower than bulk Bi_2_Te_3_ (Ma et al., 2011).

Erdogan et al. (2009) synthesized Bi_2_Te_3_ nanofilm and nanowire by electrodeposition. The acidic electrolyte containing 1 mM TeO_2_ and 1 mM Bi(NO_3_)_3_ with a pH of 1.5, in which Bi_2_Te_3_ nanofilm was deposited with a preferential orientation of (015). Additionally, the alkaline electrolyte containing 2 mM Bi(NO_3_)_3_, 1 mM TeO_2_, and 10 mM EDTA with a pH of 9.0, in which nanowire was deposited with (110) as preferential orientation. They claimed that the EDTA in the basic solution leading to the 2D growth mechanism. Furthermore, the band gap energy of Bi_2_Te_3_ nanostructures can be tuned by size and morphology of the nanostructures, as shown in the reflection absorption Fourier transform infrared spectroscopy (Erdoan and Demir, 2011).

Li et al. (2011a) electrodeposited polycrystalline Bi_2_Te_3_ nanowire arrays using AAO templates by a pulse electrodeposition method from a electrolyte containing DMSO. The results showed that the applied potential can be used to tune the composition of the nanowires. The Bi_2_Te_3_ nanowire array have a preferential orientation of (110). Additionally, Bi_2_Te_3_/Te multilayered nanowires were electrodeposited by the same method (Li et al., 2011a).

Kose et al. (2009) electrodeposited thin Bi_2_Te_3−y_Se_y_ films in the solution containing 2 mM TeO_2_, 2.5 mM Bi(NO_3_)_3_, 0.3 mM SeO_2_ and 0.1 M HNO_3_ on Au (111) at room temperature. Bi_2_Te_2.7_Se_0.3_ films was obtained at applied potential of −0.02 V vs. Ag/AgCl (3 M NaCl), which has micron-sized granular crystallites (Köse et al., 2009).

Lim et al. (2009) synthesized BiSbTe films *via* electrodeposition in the electrolyte containing 0.5 mM Bi^3+^, 32 mM SbO^+^, 2 mM HTeO_2_^+^, 0.2 M citric acid, 30 mM EDTA and 1 M HNO_3_. A Seebeck coefficient of 71 µV/K and a power factor 1.2 × 10^–4^ W/(K^2^·m) was achieved for BiSbTe films. Additionally, the amorphous Sb_2_Te_3_ films was electrodeposited at 0.01–0.03 V in the electrolyte containing 70 mM Bi^3+^, 70 mM SbO^+^, 3.5 M perchloric acid and 0.35 M tartaric acid. A Seebeck coefficient of 250 µV/K and a power factor 57 × 10^–4^ W/(K^2^·m) was achieved for Sb_2_Te_3_ films (Lim et al., 2009).

Kim et al. (2018a) electrodeposited Bi_x_Sb_2-x_Te_y_ films in the solution with 2.4 mM TeO_2_, 3.6 mM Sb_2_O_3_, 400–1,000 μM Bi(NO_3_)_3_5H_2_O, 33 mM L-tartaric acid, and 1 M HNO_3_ at fixed applied potential of −0.1 V (vs. SCE). The composition of the films were controlled by [Sb]/[Bi] ratio. The results showed that the substitution of Bi with Sb would improve the mobility, while suppress the carrier concentration. The deposited Bi_10_Sb_30_Te_60_ film has a high Seebeck coefficient, which results in a power factor (PF) of ∼490 μW/m K^2^ (Kim et al., 2018a).

Ma et al. (2010) electrodeposited Bi_1-x_Sb_x_ and Bi_2-x_Sb_x_Te_3_ thin films at 25°C with different morphologies, such as thin sheets, rods, dendrites, and spherical particles. The Bi_1-x_Sb_x_ film was deposited in the electrolyte containing 2 mM Bi(NO_3_)_3_, 1 mM SbCl_3_, 0.2 M C_4_H_6_O_6_, and 0.1 M HNO_3_. Additionally, The Bi_2-x_Sb_x_Te_3_ film was deposited in the electrolyte containing 0.3 mM TeO_2_, 0.2 mM Bi(NO_3_)_3_, 1 mM SbCl_3_, 0.2 M C_4_H_6_O_6_, and 0.1 M HNO_3_. Furthermore, the results indicated that the underpotential deposition mechanism would lead to the formation of (Bi_0.5_Sb_0.5_)_2_Te_3_, however the overpotential deposition would result in the formation of Bi_0.5_Sb_1.5_Te_3_. Meanwhile different deposition mechanism can be triggered by applied potential (Ma et al., 2010).

Jin and Wang (2010) electrodeposited n-Type thin Bi_2_Te_3-y_Se_y_ films using Au, Bi, and Bi_2_Te_3-y_Se_y_ as substrates. The electrolyte contained 8 mM HTeO_2_^+^, 8 mM Bi^3+^, 1 mM H_2_SeO_3_, and 1 M HNO_3_. The substrates have significant effect on the morphology of films, as well as the crystal orientation. The preferred orientation of (015) with rhombohedral structure was obtained when using Bi_2_Te_3-y_Se_y_ as substrate. Additionally, the films deposited on the Bi_2_Te_3-y_Se_y_ substrate showed the highest power factor after annealing (Jin and Wang, 2010).

Golgovici et al. (2011) investigated electrodeposition of Bi_2_Te_3_, Sb_2_Te_3_, BiSb, and BiSbTe films in an aqueous solution containing 5 M NaCl and 1 M HCl or an ionic liquid with choline chloride and malonic acid mixture. The concentrations of Bi, Sb and Te ion were controlled between 10 and 90 mM. Morphology and composition of BiSbTe was modified by increasing the current pulses (Golgovici et al., 2011).

Liu et al. electrodeposited Bi_2_Te_3_ pillars using multi-channel glass molds as template. The results showed that pulsed electrodeposition method is helpful to achieve high aspect ratio filling. The n-type Bi_2_Te_3_ arrays with aspect ratio exceeding ten was obtained at a pulse circle of −0.2 V for 4 s, +0.5 V for 1 s, and 0 mV for 3 s (vs. SCE). The precursor concentration in the electrolyte includes 7.5 mM Bi^3+^ and 10 mM HTeO_2_^+^. Furthermore, the electrical conductivity of as-deposited Bi_2_Te_3_ pillars is the same magnitude as bulk Bi_2_Te_3_ (Liu and Li, 2011).

Li et al. (2011b) synthesized heterogeneous thermoelectric nanowire arrays of multilayer Bi_2_Te_2_Se/Te using template direction electrodeposition. The thickness of the Te section can be modulated by tailoring Te ion concentration. The diameter of the heterogeneous nanowires is from 60 to 85 nm. Additionally, the Bi_2_Te_2_Se segment can change to Bi_2_Se_2_Te by lowing the Te ion concentration to a certain level (Li et al., 2011b).

Pinisetty et al. (2011a) fabricated polycrystalline Bi_2_Te_3_ nanowires and nanotubes arrays by electrodeposition. The applied potential had effect on the composition, where both Bi-rich (p-type)and Te-rich (n-type) nanowires or nanotubes can be deposited. The lamellar thickness of bath morphologies were about 17–24 nm. The nanowires and nanotubes had a Seebeck coefficient of 11.5 and 17 μV/K, respectively, which were deposited at −0.4 V. However, when applied potential was −0.065 V, Seebeck coefficient of −48 and −63 μV/K were obtained for the nanowires and nanotubes, respectively (Pinisetty et al., 2011a).

Lim et al. (2012a) synthesized Bi_x_Sb_2-x_Te_3_ films by electrodeposition in an electrolyte containing 0.8 mM TeO_2_, 0.2 mM Bi(NO_3_)_3_, 0.8 mM Sb_2_O_3_, 33 mM tartaric acid, and 1 M HNO_3_. The composition of the thin films can be controlled by applied potential, where stoichiometry can be achieved from −0.10 to −0.15 V vs. SCE. Additionally, at more negative applied potential, the thermoelectric property of Bi_x_Sb_2-x_Te_3_ films was degraded, which might owing to higher defect density. The electrical and thermoelectric properties can be enhanced by annealing in reducing environment (Lim et al., 2012a).

Peranio et al. (2012) synthesized Bi_2_Te_3_ nanowires by a potential-pulsed electrodeposition using AAO as template in a solution with 15 mM HTeO_2_^+^, 10 mM Bi^3+^ and 1 M HNO_3_. The nanowires had a stoichiometric composition with diameters of 50–80 nm and a length of 56 μm. The nanowires are single-crystalline with no grain boundaries. The XRD pattern revealed that growth direction of the nanowires were (110) and (210). Additionally, the c axis of the Bi_2_Te_3_ crystal was perpendicular to nanowire axis (Peranio et al., 2012).

Frantz et al. (2012) electrodeposited bismuth telluride nanowires from an electrolyte with1.5 mM Bi^3+^, 15 mM HTeO_2_^+^ and DMSO using polycarbonate as template. The DMSO would shift the reduction potential to more negative side and inhibit the cation diffusion. The nanowires deposited -0.1 V vs Ag/AgCl at have a diameter of 60 nm diameter with stoichiometric composition. The crystal structure of the nanowires was polycrystalline with a preferential orientation perpendicular to the (015) planes (Frantz et al., 2012).

Ma et al. (2012a) synthesized thin Sb_2_Te_3_ and Bi_2_Te_3_ films using goldthe Au-coated silicon as substrate in an acidic bath with Bi(NO_3_)_3_·5H_2_O, TeO_2_, Sb_2_O_3_, 1 M HNO_3_ and 0.5 M tartaric acid at room temperature by electrochemical deposition. The as-deposited Bi_2_Te_3_ films were polycrystalline, but the Sb_2_Te_3_ films were amorphous. Additionally, the Sb_2_Te_3_ films showed both Sb_2_Te_3_ and Te phase after annealing (Ma et al., 2012a).

Zhu et al. (2008) electrodeposited p-type quaternary thin BiSbTeSe films using Au as substrate in a acidic solution with 0.5 mM Se(IV), 12 mM Te(IV), 2.5 mM Bi(III), 10 mM Sb(III), 0.67 M tartaric acid at room temperature. The thickness of the films was controlled to 8 μm. The applied potential can be used to tailoring the composition of the films. The as-deposited films were amorphous, however they changed to polycrystalline after annealing based on the XRD patterns. A maximum power factor of 620 µW/(K^2^·m) was achieved for the thin BiSbTeSe films after post-annealing with Seebeck coefficients of 116–133 μV/K (Zhu and Wang, 2012).

Banga et al. (2012) fabricated Bi_2_Te_3_/Bi_2−x_Sb_x_Te_3_ heterostructure by pulsed potentiostatic electrodeposition method. The solution consisted Na_2_TeO_3_, Bi(NO_3_)_3_, Sb(III), 2 M nitric acid, and 0.3 M tartaric acid. The heterostructure had a layer periodicity in the range of 10–30 nm. The XRD data showed that the multilayer films possessed a (015) texture (Banga et al., 2012).

Zhu et al. (2008) synthesized n-type Bi_2_Te_3-y_Se_y_ films using ITO-coated glass as substrates in the acidic solution containing 10.0 mM HTeO_2_^+^, 7.5 mM Bi^3+^, 1.1 mM SeO_3_^2-^ and 0.5 M HNO_3_ at room temperature by pulsed electrodeposition. The smooth and compact Bi_2_Te_3-y_Se_y_ films were obtained. Increasing the cathodic current density would decrease the grain size of the films. The Bi_2_Te_3-y_Se_y_ films had a Seebeck coefficient of about -92 μV/K and electrical resistivity of about 109.4 μΩ m (Zou et al., 2012).

Naylor et al. (2012) synthesized Bi_2_Te_3_ films with stoichiometric composition Bi_2_Te_3_ in the electrolyte consisting of 10 mM Te(IV), 7.5 mM Bi(III), sodium lignosulfonate (SL) and 1 M HNO_3_. The sodium lignosulfonate acted as a surfactant, which would improve morphology and roughness of the Bi_2_Te_3_ films and achieve better alignment in the (110) plane. The optimal concentration of SL is from 60 to 80 mg/L at a deposition potential of -0.1 V vs SCE (Naylor et al., 2012).

Limmer et al. (2012) reported the electrodeposition of 75 nm diameter nanowire arrays with a composition of Bi_2_(Te_0.95_Se_0.05_)_3_ onto Si substrates using AAO as template in the electrolyte containing 80 mM Bi(NO_3_)_3_•5H_2_O, 40–80 mM TeCl_4_, 0.8–1.2 mM SeO_2_ and 0.1 M KClO_4_ in dimethyl sulfoxide. The nanowires are polycrystalline with grain size of 5–10 nm (Limmer et al., 2012).

Ma et al. (2012b) synthesized ternary compounds (Bi_x_Sb_1-x_)_2_Te_3_ and Bi_2_(Te_1-y_Se_y_)_3_ by electrodeposition using gold-coated silicon as substrates in the electrolyte with TeO_2_, Bi(NO_3_)_3_·5H_2_O, SbCl_3_ and Na_2_SeO_3_, 1 M HNO_3_ and 0.67 M tartaric acid at room temperature. The p-type (Bi_x_Sb_1-x_)_2_Te_3_ films had the highest power factor obtained with composition close to Bi_0.5_Sb_1.5_Te_3_ deposited at a relatively large negative potential. In addition, Bi_2_(Te_1-y_Se_y_)_3_ thin films showed n-type behaviors with composition close to Bi_2_Te_2.7_Se_0.3_ (Ma et al., 2012b).

Fu et al. (2013) fabricated Ag/Bi_2_Te_3_ multilayer nanowires by pulse electrochemical deposition using AAO as the template in the electrolyte consisted of 0.1 M HTeO_2_^+^, 75 mM Bi(NO_3_)_3_, 10 mM AgNO_3_, and 1 M HNO_3_. The deposited the Bi_2_Te_3_ had a rhombohedral lattice phase and Ag had a cubic lattice phase. The length of each layer ranged from 25 to 45 nm (Fu et al., 2013).

Nguyen et al. (2012) investigated the electrodeposition of Bi_2_Te_3_ film in the electrolyte consisting of 50 mM of 50 mM TeCl_4_, Bi(NO_3_)_3_, 0.5 M lithium nitrate, and ethylene glycol. The results showed that the electrochemical reduction reaction of both Bi^3+^ and Te^4+^ ions were carried out at applied potential more negative than 0.2 and 0.55 V vs. SHE, and the reaction is one step without the formation of intermediates. The Bi and Te ions had the similar diffusion coefficients and the reaction rate constants. Bi_2_Te_3_ films stoichiometric composition were deposited at current densities up to 5 A/dm^2^ (Nguyen et al., 2012).

Agapescu et al. (2013) electrodeposited of Bi, Te, and Bi_2_Te_3_ films in an ionic liquids consisting of 10 mM BiCl_3_ and TeO_2_, choline chloride, and oxalic acid (ChCl–OxA) at 60°C.

Kim and Oh (2013) fabricated a thermoelectric device using n-type Bi_2_Te_3_ and p-type Sb_2_Te_3_ thin films as basic element legs. The device has a cross-plane configuration with 242 pairs of legs by flip-chip bonding of top electrodes. The thickness of both Bi_2_Te_3_ and Sb_2_Te_3_ films were about 20 μm. Additionally, the n-type Bi_2_Te_3_ and p-type Sb_2_Te_3_ films showed Seebeck coefficients of -59 μV/K and 485 μV/K, respectively. Furthermore, an open-circuit voltage of 0.294 V and a maximum output power of 5.9 μW were achieved at a temperature difference of 22.3 K (Kim and Oh, 2013).

Manzano et al. (2013) electrodeposited Bi_2_Te_3_ films with preferentially oriented of (110) direction in the electrolyte containing 10 mM HTeO_2_^+^, 7.5 mM Bi^3+^ and 1 M HNO_3_ at applied potential of 0.02 V vs. Ag/AgCl on a Pt substrate. When using pulsed electrodeposition method, the results indicated that at a pulse of on-time = off-time = 0.1 s the films achieved a Seebeck coefficient of −72 μV/K and power factor of 440 μW/(K^2^·m), which is measured at 107°C. Additionally, when using potentiostatic method, a Seebeck coefficient of -73 μV/K at 107°C and power factor of 600 μW/(K^2^·m) was obtained at 107°C (Manzano et al., 2013).

Zhou et al. (2013) electrodeposited n-type phosphorus-doped Bi_2_Te_3_ films on a stainless-steel electrode in the solution containing 10 mM TeO_2_, 8 mM Bi(NO_3_)_3_, 4 mM H_3_PO_4_ and 1 M HNO_3_. The as-prepared films had the thermal conductivity of 0.47 W/(mK) and the electrical conductivity of 280 S/cm (Zhou et al., 2013).

Rashid et al. (2013) synthesized Bi_2_Te_3_ films by galvanostatic electrodeposition in a solution containing 8 mM HTeO_2_^+^, 8 mM Bi^3+^ and 1 M nitric acid. The results indicated that annealing process would enhance the carrier mobility while suppressing the carrier concentration. Additionally, the Seebeck coefficient can be enhanced from -57 to -169.49 µV/K and the power factor can be enhanced from 2.74 to 1737 µW/(K^2^·m) by post annealing process for p-type Bi_2_Te_3_ film. Moreover, the Seebeck coefficient can be improved from 28 to 112.3 µV/K and the power factor can be improved from 2.57 to 443 µW/(K^2^·m) by post-annealing process (Rashid et al., 2013).

Cao et al. (2013) fabricated thin Bi_2_Te_3_ films by electrodeposition in the solution with 10 mM HTeO_2_^+^, 8 mM Bi^3+^ and 1 M HNO_3_ at room temperature. The substrates used during the deposition had an epitaxial seed layer, which would help to reduce the lattice mismatch between Bi_2_Te_3_ and Silicon. Moreover, more uniform structure and better crystallinity can be achieved. Both doped and intrinsic silicon were used as substrate, while the results showed that a more compact thin Bi_2_Te_3_ film with preferential orientation of (001) was obtained for intrinsic silicon, which also showed better thermoelectric performance and smoother surface morphology. Compared to the thin film with preferential orientation of (110), the electrical conductivity is about 72% higher and the power factors is about 45% higher. Additionally, the electrical conductivity and Seebeck coefficient was suppressed by reducing the seed layer thickness from 40 to 20 nm, which can be attributed to the insufficient charge transfer during electrodeposition (Cao et al., 2013).

Wu et al. (2013) investigated the effect of chloride on the electrodeposition of Bi_2_Te_3_ films in the solution containing TeCl_4_, Bi(NO_3_)_3_·5H_2_O and ethylene glycol. The results indicated that the presence of chloride could enhance the reduction reaction rate of Te significantly, where the reaction rate with chloride in the solution is three orders of magnitude higher than the rate without chloride. Additionally, Bi_2_Te_3_ films with stoichiometric composition and smooth morphology were electrodeposited in certain potential window. A Seebeck coefficient of -120 µV/K was achieved for the Bi_2_Te_3_ films (Wu et al., 2013).

Yoo et al. (2013a) electrodeposited Bi_x_Te_y_ thin films from nitric acid baths with 2.5–10 mM Bi(NO_3_)_3_, 10 mM TeO_2_, and 1.5 M HNO_3_ using Au/Ni/Si as substrates. The films with surface morphologies of granular and needle-like structures were deposited at different Te content. Higher of Bi ions concentration in electrolytes would result in higher power factor. Additionally, the power factor was not improved significantly owing to the interdependence of the electrical conductivity and the Seebeck coefficient (Yoo et al., 2013a).

Wang et al. (2013) synthesized Bi_2_Te_2.7_Se_0.3_ and Bi_0.5_Sb_1.5_Te_3_ by electrodeposition combined with post annealing. The solution to electrodeposit n-type Bi_2_Te_2.7_Se_0.3_ contained 8 mM HTeO_2_^+^, 8 mM Bi^3+^, 1 mM H_2_SeO_3_ and 1 M HNO_3_, while the electrolyte to electrodeposit p-type Bi_0.5_Sb_1.5_Te_3_ contained 2 mM Bi^3+^, 10 mM HTeO_2_^+^, 100 mM Sb(III) and 1 M HNO_3_. The as-deposited films possess amorphous structure and can be transferred to nanocrystalline after annealing. The annealed films show a preferred orientation of (015). The maximum power output of 77 μW was achieved with open-circuit voltage of 660 mV with a temperature difference of 20 K at 25°C. Additionally, a power density of 770 μW/cm^3^ was obtained (Wang et al., 2013).

Rashid et al. (2013) synthesized n-type Bi_2_Te_3_ films with a prominent orientation of (110) in the acidic solution with TeO_2_ and Bi(NO_3_)_3_ on gold electrode. The Bi_2_Te_3_ films are nanocrystalline with grain size ranged from 21 to 45 nm. The results showed that the electrodes distance could be used to tune electrical and thermoelectric properties of the films, thus improving carrier charge mobility without varying of the Seebeck coefficient and carrier concentration. The highest power factor of 820 μW/K^2^·m was achieved with an electrical conductivity of 2.13 × 10^3^ S/cm and Seebeck coefficient of -61.2 μV/K (Rashid and Chung, 2013).

Yoo et al. (2013b) synthesized Bi_x_Sb_2-x_Te_3_ films use potentiostatic electrodeposition method at room temperature in an acidic electrolyte containing 0.8 mM TeO_2_, 0.2 mM Bi(NO_3_)_3_, 0.8 mM Sb_2_O_3_, 1 M HNO_3_, and 33 mM tartaric acid. When the applied potential was controlled between -0.10 and -0.15 V versus SCE, thin films with composition near stoichiometric were deposited. Additionally, reducing the applied potentials would result in suppressing the electrical and thermoelectric properties, probably owing to higher defect density (Yoo et al., 2013b).

Ng et al. (2014) fabricated the binary Bi_2_Te_3_ and ternary BiSbTe nanowires using template (AAO) directed electrodeposition method in a solution compose of 10 mM TeO_2_, 20 mM Bi(NO_3_)_3_·5H_2_O and 1 M HNO_3_,. The results showed that reducing the applied potentials can increase the Sb composition, while increasing the applied potentials would facilitate the formation of Bi_2_Te_3_ (Ng et al., 2014).

Zou et al. (2014) investigated electrodeposition of n-type Bi_2_Te_3-y_Se_y_ film in the solution containing 10.0 mM HTeO_2_^+^, 1.1 mM SeO_3_^2-^, 7.5 mM Bi^3+^, and 1 M HNO_3_ at room temperature. The nucleation and growth mechanism were examined. The electrochemical reaction rate was controlled by diffusion and irreversible with the limiting current density of 1.78 mA/cm^2^. A flocculent film was deposited when the applied potential was larger than limiting current without agitation. However, Bi_2_Te_3-y_Se_y_ film with smooth morphology was deposited at 4 mA/cm^2^ with agitation. Bi_2_Te_3-y_Se_y_ film deposited at 1 mA/cm^2^ have relatively high power factor and electrical conductivity (Zou et al., 2014).

Maas et al. (2014) electrodeposited Bi_2_Te_3_ in acidic solution with 20 mM HTeO_2_^+^ and 20 mM Bi^3+^. The anode is Bi_2_Te_3_ as a sacrificial the source of cations. A homogeneous Bi_2_Te_3_ film with a thickness of 300 µm was deposited using Bi_2_Te_3_ as anode, while without Bi_2_Te_3_ as anode the thickness can be obtained is 10 times thinner. A power factor of 500 µW/(K^2^·m) was achieved (Maas et al., 2014).

Szymczak et al. (2014) electrodeposited n-type Bi_2_Te_3_ films in an ionic liquid with 1-ethyl-1-octyl-piperidinium bis(trifluoromethylsulfonyl)imide (EOPipTFSI) and 1-ethyl-1-octyl- piperidinium bromide (EOPipBr). The atomic ratio of EOPipTFSI and EOPipBr is 95:5. According to the result, this ionic liquid is stable at high cathodic applied potential, which provide a larger window to deposited Bi_2_Te_3_ compound. The morphology of the Bi_2_Te_3_ film can be tuned by precursor concentration, in which mirror-like films can be deposited with good uniformity. Additionally, an electrical resistivity of 133 µΩ m and Seebeck coefficient of -70 µV/K were achived (Szymczak et al., 2014).

Jiang et al. (2014) fabricated Bi_2_Te_3_/PEDOT:PSS/Bi_2_Te_3_ composite film by electrodeposition of Bi_2_Te_3_ onto poly (3,4-ethylenedioxythiophene): poly (styrenesulfonate) (PEDOT:PSS) film. The solution contained TeO_2_, Bi(NO_3_)_3_, and 1 M HNO_3_. A thermal conductivity of 0.169–0.179 W/(mK) was obtained. ZT value of 1.72 × 10^–2^ was achieved for Bi2Te3/PEDOT:PSS/Bi_2_Te_3_ composite film with electrical conductivity of 403.5 S/cm (Jiang et al., 2014).

Caballero-Calero et al. (2014) electrodeposited Bi_2_Te_3_ films in a solution with 10 mM HTeO_2_^+^, 7.5 mM Bi^3+^, 1 M HNO_3_. The Bi_2_Te_3_ films have a preferred orientation of (110) with c-axis parallel the substrate. Additionally, the effect of sodium lignosulfonate as surfactant on morphology was examined. Seebeck coefficient was determined to be -80 ± 6 µV/K (Caballero-Calero et al., 2014).

Wu et al. (2014) electrodeposited SbBi, Sb_2_Te_3_, and BiSbTe alloys in the electrolyte containing TeCl_4_, SbCl_3_, Bi(NO_3_)_3_, and ethylene glycol. The electrochemical reaction mechanism of Sb in chloride-free ethylene glycol was investigated. The results showed that the diffusion coefficients of Sb(III), Te(IV) and Bi(III) were comparable in ethylene glycol. Additionally, the onset potential of Sb is more negative than that of Te. During the electrodeposition of BiSbTe alloys, BiTe was deposited first followed by increase of Sb composition at more negative applied potential. (Wu et al., 2014).

Patil et al. (2015) electrodeposited fern shaped Bi_2_Te_3_ thin film in the solution containing 10 mM Te(IV), 7 mM Bi(NO_3_)_3_, and 1 M HNO_3_.

Matsuoka et al. (2015) electrodeposited Bi_2_Te_3_/Bi_2_Se_3_ multiplayer heterostructure in two baths sequentially. The layer thickness was fixed to about 1 µm and the number of layers were varied from 2 to 10. The deposited multilayer structure is n-type with nanocrystalline. The boundaries between different layers were not clear planar. The number of the layers had a dramatic effect on the electrical conductivity, where more layers resulted in higher electrical conductivity, while Seebeck coefficient remained unchanged. The 10-layer Bi_2_Te_3_/Bi_2_Se_3_ heterostructure has a power factor of 144 µW/(K^2^∙m), which is about 3 times higher than that of the 2-layer heterostructure (Matsuoka et al., 2015).

Caballero-Calero et al. (2015) electrodeposited Bi_2_Te_3-y_Se_y_ films in a conventional three electrode cell in the solution containing 9 mM HTeO_2_^+^, 7.5 mM Bi^3+^, 1 mM H_2_SeO_3_, and 1 M HNO_3_. The influence of additives (*i.e.,* sodium signosulfonate (SLS) and EDTA) in morphology, stoichiometry, structure and Seebeck coefficient was studied. The films synthesized with SLS had high crystallographic orientation and better morphology, while films deposited in the presence of EDTA had higher content of bismuth. The combination of both additives would improve the quality of stoichiometric Bi_2_Te_2.7_Se_0.3_ films, namely denser morphology, higher orientation and higher Seebeck coefficients (60% larger) when compared with films deposited without additives. (Caballero-Calero et al., 2015).

Zhou et al. (2015) synthesized Bi_2_Te_3_ thin films by the pulsed electrodeposition method in the solution consisting of 40 mM HTeO_2_^+^, 30 mM Bi^3+^ and 1.7 M HNO_3_. The effect of deposition parameters on the composition and microstructure was investigated. The results indicated that the stoichiometry and morphology can be improved by a large pulse off-to-on ratio with a pulsed applied potential of 0 mV vs. Ag/AgCl. Additionally, larger pulse off-to-on ratio would enhance the ZT of Bi_2_Te_3_ films owing to suppressing the thermal conductivity and improving the Seebeck coefficient. The highest ZT value was 0.16 obtained at a pulse off-to-on ratio of 50 (Zhou et al., 2015).

Li et al. (2015) reported the electrodeposition of BiSbTe nanowires in the electrolyte containing 15 mM HTeO_2_^+^, 40 mM SbO^+^, 2 mM Bi^3+^, 0.3 M tartaric acid and 1 M HNO_3_. Their data showed that the pulse electrodeposit method would help to improve the uniformity and crystallinity of Bi_0.5_Sb_1.5_Te_3_ nanowires, which resulted in higher electrical and thermal conductivity, compared to the direct current deposited nanowires. Additionally, the pulse electrodeposit method would also enhance the Seebeck coefficient of nanowires, which was attributed to a more homogeneous distribution of the elements. The highest ZT value was 1.14 at 330 K achieve by pulse-deposited Bi_0.5_Sb_1.5_Te_3_ nanowires (Li et al., 2015).

Song et al. (2015) synthesized thin Bi_2_Te_3_ films in a acidic bath with 7.5 mM Bi(NO_3_)_3_, 10 mM TeO_2_, cetyltrimethylammonium bromide (CTAB) and 1.5 M HNO_3_ at room temperature. CTAB acted as a surfactant. The results indicated that the presence of CTAB would help to improve the surface morphology and mechanical properties Bi_2_Te_3_ films. However, the electrical and thermoelectric properties were preserved (Song et al., 2015).

Uda et al. (2015) fabricated Bi_2_Te_3_ thermoelectric micro-device by electrodeposition in an electrolyte composing of Bi(NO_3_)_3_·5H_2_O, TeO_2_, and HNO_3_. The size effect of electrode was examined. The cross-section of the TE units is 50 × 50 μm^2^ with depth of 20 µm. Additionally, the device had a eight arrays, which composed of 110 TE units. A maximum power output of 0.96 µW was achieved with an open-circuit voltage of 17.6 mV (Uda et al., 2015).

Chang et al. (2015) examined the electrodeposition of individual n-type Bi_2_Te_3_ nanowires (NWs) using polycarbonate membranes (PCM) as templates in the solution 10 mM HTeO_2_^+^, 15 mM Bi^3+^, 1 M HNO_3_ and 50 v/v % DMSO. The electrodeposition conditions, such as the applied potential can be used to control the composition of Bi_2_Te_3_. Additionally, increase the Te composition would increase the average grain size of NWs, as well as the electrical conductivity. The maximum power factor of 195.8 µW/(mK^2^) was achieved at 300 K for the Te-rich NW with diameter of 162 nm (Chang et al., 2015).

Shin and Oh (2015) fabricated a thermoelectric device based on thin film by combining electrodeposition and the flip-chip process. The thermoelectric materials used in the device are the n-type Bi_2_Te_3_ and p-type Sb_2_Te_3_ thin film, which is deposited on Ti/Cu/Au substrate in the solutions with 25 mM Bi ion, 25 mM Te ion and 1 M HNO_3_ for Bi_2_Te_3_ and 63 mM Sb ion, 7 mM Te ion. The device with 242 pairs thermoelectric legs have a internal resistance of 21.4 Ω, which have a output voltage of 320 mV and output power of 1.1 mW at 39.7 K temperature difference. Additionally, the calculated power density of 3.84 mW/cm^2^ (Shin and Oh, 2015).

Abellán et al. (2015) synthesized thin Bi_2_Te_3_ films containing TeCl_4_, Bi(NO_3_)_3_ and dimethyl sulfoxide. Different substrates were used, such as CdTe/FTO and SnO_2_:F coated glasses. Additionally, the deposits films were n-type semiconductors with trigonal crystal structure and stoichiometric composition dimethyl sulfoxide (Abellán et al., 2015).

Kulsi et al. (2015) synthesized thin Bi_2_Te_3_ films with preferred crystal orientation of (018) in the solution consisting of 15 mM TeO_2_ and 10 mM Bi (NO_3_)_3_. The effect of different surfactant on the morphology was examined, including sodium dodecyl sulfate (SDS) and polyvinylpyrrolidone (PVP). The results indicated that improving the surface morphology would help to enhancing the carrier mobility. A ZT value of 0.28 was achieved using SDS as surfactant, which was measured at room temperature (Kulsi et al., 2015).

Şişman and Başoğlu (2016) fabricated thin Bi_2_Te_3-y_Se_y_ films by electrodeposition in the solution containing 2 mM TeO_2_, 2.5 mM Bi(NO_3_)_3_, SeO_2_ and 0.1 M HNO_3_ with Au as substrate. The Se composition was controlled to be 0.3 to 2.5. The results showed that replacement of Te by Se atoms would push the XRD diffraction peaks positions Bi_2_Te_3-y_Se_y_ to higher angle, which is attributed to the change of crystal lattice constant (Şişman and Başoğlu, 2016).

Lei et al. (2016a) synthesized of 600 μm-thick n-type Bi_2_Te_3_ films by pulsed and potentiostatic electrodeposition in the electrolyte consisting of 70 mM TeO_2_, 52.5 mM Bi^3+^, 2 M nitric acid and polyvinyl alcohol (PVA). The results indicated that compact and uniform Bi_2_Te_3_ films was electrodeposited which composition near stoichiometric and hexagonal crystal structure. Moreover, the film growth can reach 100 μm/h. Additionally, a Seebeck coefficient of -200 µV/K and an electrical conductivity of 400 S/cm were achieved, resulting in a power factor of 1.6 × 10^3^ µW/(mK^2^) (Lei et al., 2016a).

Yang et al. (2016) electrodeposited p-type BiSbTe thin films using ITO glasses as substrate in the electrolyte composing of 2 mM TeO_2_, 0.5 mM Bi_2_O_3_, 3.5 M HClO_4_, 1 M HNO_3_ and 0.35 M C_4_H_6_O_6_. The Sb^3+^ concentration and current density were the variables during the electrodeposition. Thin BiSbTe films showed different morphologies, such as ball-type, mixed-type and acicular-type. The Seebeck coefficient of 32.89 μV/K was obtained (Yang et al., 2016).

Patil et al. (2016) electrodeposited thin Bi_2_Te_3_ film in a solution with 10 mM Te(IV), 7 mM Bi(NO_3_)_3_·5H_2_O and 1 M HNO_3_. The XRD pattern showed that the Bi_2_Te_3_ film was nanocrystalline with grain size of 18.08 nm and had a preferred orientation of (015) with rhombohedral crystal structure (Patil et al., 2016).

**FIGURE 6:**
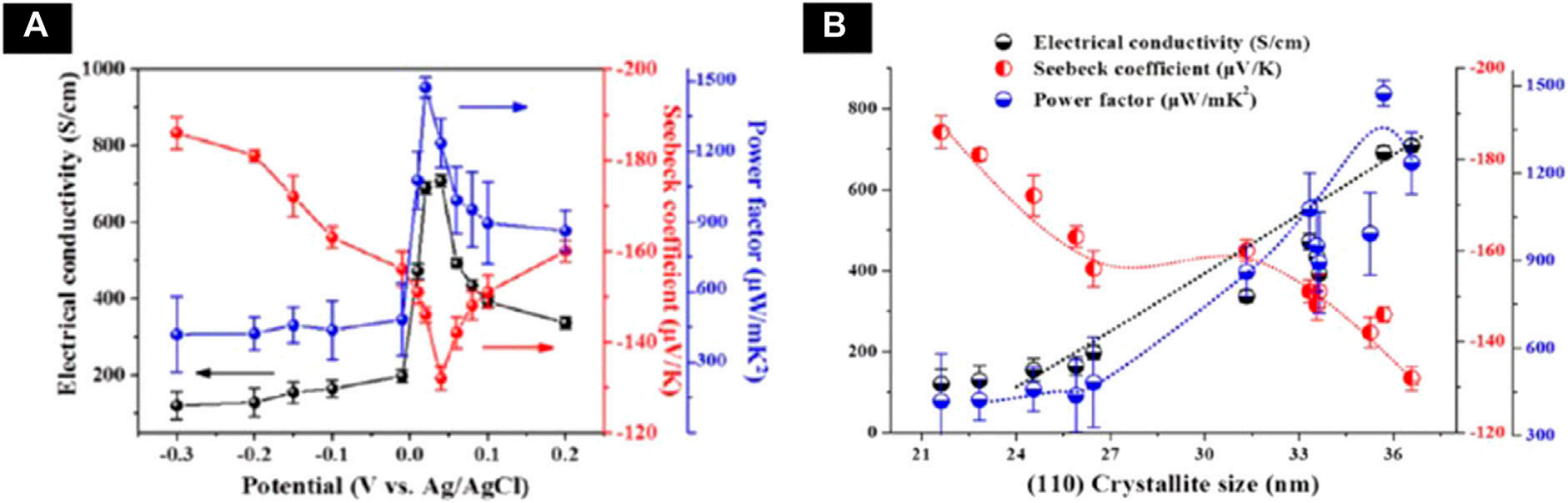
**(A)** Corelation of electrical conductivity (black circle), Seebeck coefficient (red circle), and power factor (blue circle) with different applied potentials (V vs Ag/AgCl) **(B)** Corelation of the electrical conductivity (black), Seebeck coefficient (red), and power factor (blue) with (110) crystallite size (Na et al., 2016).

Na et al. (2016) electrodeposited n-type Bi_2_Te_3_ films in the electrolyte with 10 mM HTeO_2_^+^, 8 mM Bi^3+^ and 1 M HNO_3_ on a flexible substrate. The effect of applied potential on the crystal structure and thermoelectric properties were systematically studied. The Bi_2_Te_3_ film with preferred orientation of (110) is deposited. The highest power factor of 1,473 μW/(K^2^·m) was achieved for the film electrodeposited at applied potential of 0.02 V with electrical conductivity of 691 S/cm. The effect of applied potential and grain size on the electrical and thermoelectric properties were shown in Figure 6. A flexible thermoelectric device was fabricated using Bi_2_Te_3_ as n-type material and poly (3,4-ethylene dioxythiophene)s as p-type material. The generator achieved a output voltage of 5 mV and output power of 56 nW with temperature difference of 12 K (Na et al., 2016).

**FIGURE 7:**
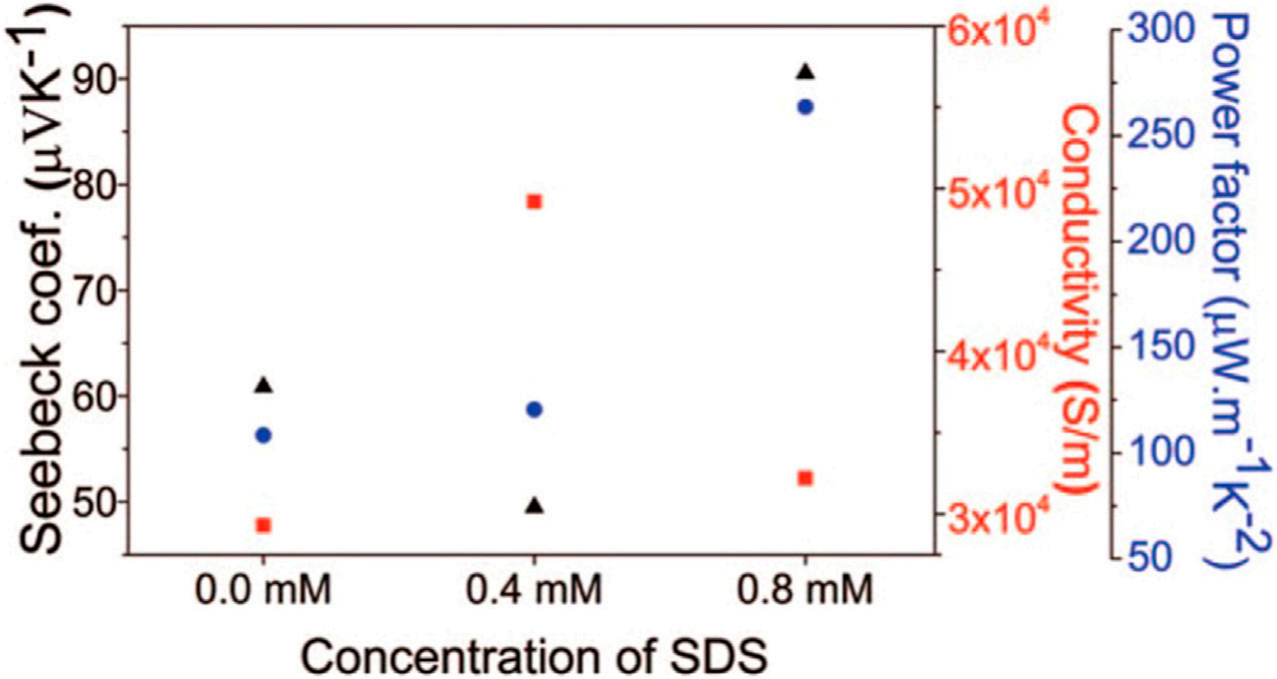
Electrical resistivity (square), Seebeck coefficient (triangle), and power factor (circle) of annealed films deposited with different concentrations of SDS (Lal et al., 2017).

Lal et al. (2017) synthesized p-type (Bi_x_Sb_1-x_)_2_Te_3_ thin films using pulsed electrodeposition in the electrolyte consisting of 15 mM HTeO_2_^+^, 5 mM Sb_2_O_3_, 5 mM Bi(NO_3_)_3_, 0.2 M tartaric acid, sodium dodecyl sulfate (SDS) and dimethyl sulfoxide. The results indicated that the present of SDS would improve the Seebeck coefficient and power factor of the films as shown in Figure 7. (Lal et al., 2017).

Additionally, many other groups reported the results of characterization of BiTe/Se electrodeposits based on various experimental conditions which are summarized on Table 1. (Jagadish et al., 2015; Lei et al., 2016b; Wu et al., 2016b; Hasan et al., 2016; Kulsi et al., 2016; Manzano et al., 2016; Wu et al., 2017b; Kang et al., 2017; Lal et al., 2017; Lei et al., 2017).

### Electrodeposition of Bi_x_Sb_y_ Based Materials

Dou et al. (2008) synthesize Bi/BiSb superlattice nanowires by template-directed electrodeposition method, in which AAO was used as template. The electrolyte for electrodeposition contains a mixture of 80 mM SbCl_3_, 40 mM BiCl_3_, 50 g/L citric acid, 40 g/L tartaric acid, 70 g/L NaCl, 100 g/L glycerol and 1.0 M HCl at pH value of 0.82 (Dou et al., 2008).

Weber et al. (2008) electrodeposited high density nanowire arrays in AAO templates from the electrolyte of 50 mM Bi^3+^ + 50 mM Sb^3+^ in dimethyl sulfoxide.

Dou et al. (2009) synthesized Bi/BiSb multilayer nanowires by pulsed electrodeposition with small bilayer thickness. The electrolyte for the deposition contained a mixture of 80 mM SbCl_3_, 40 mM BiCl_3_, 0.24 M citric acid, 0.27 M tartaric acid, 1.2 M NaCl, 0.1 M glycerol and 1.0 M HCl. Additionally, the modulating time was used to control the segment length and layer thickness of the nanowires (Dou et al., 2009).

Muller et al. (2012) synthesized Bi_1−x_Sb_x_ nanowires with Sb composition in the range from 0.05 to 0.40 and diameter in the range from 20 to 100 nm. The results showed that applied potential and ratio of Bi/Sb ions would influence the composition of Bi_1−x_Sb_x_ nanowires (Müller et al., 2012).

Limmer et al. (2015) electrodeposited Bi_x_Sb_y_ in the non-aqueous baths consisting of Sb salts, Bi(NO_3_)_3_·5H_2_O, dimethyl sulfoxide and KClO_4_. The effect of different Sb salts on the crystalline quality and preferred orientations were investigated. The results showed that nanowire arrays synthesized with SbI_3_-based bath were polycrystalline. However, nanowire arrays synthesized with SbCl_3_-based bath have a trigonal orientation, and composition of these nanowires remained constant along the nanowires. Additionally, there was a composition gradient along the radius of the nanowires array, where nanowires of Bi_0.75_Sb_0.25_ were obtained in the center area and nanowires of Bi_0.70_Sb_0.30_ were obtained in the edge region (Limmer et al., 2015).

### Electrodeposition of Bi_2_Se_3_ Based Materials

Xiao et al. (2009) electrodeposited thin Bi_2_Se_3_ films using Pt as substrate by atomic layer epitaxy. The electrochemical reaction mechanism of Bi and Se alone were investigated by cyclic voltammetry. The electrodeposition mechanism Bi_2_Se_3_ is underpotential deposition (UPD). The synthesized Bi_2_Se_3_ films had an orthorhombic structure with stoichiometric composition. Additionally, the bandgap of Bi_2_Se_3_ films the was 0.35 eV. (Xiao et al., 2009).

Li et al. (2010c) synthesized Bi_2_Se_3_ thin films by electrodeposition in the solution containing SeO_2_, Bi(NO_3_)_3_, and HNO_3_ using Ti and indium tin oxide-coated glass as substrates at room temperature. The results indicated that the substrate had dramatic effect on the crystal structure of Bi_2_Se_3_ thin films. Pure rhombohedral crystal structure was obtained on the indium tin oxide-coated glass substrate, while both rhombohedral and orthorhombic crystal structure was obtained on Ti (Li et al., 2010c).

Xue et al. (2014) fabricated Bi_2_Se_3_/Bi multilayered nanowire arrays by pulsed electrodeposition using AAO as template in the electrolyte with 7.5 mM H_2_SeO_3_, 25 mM Bi^3+^ and 7 mM HNO_3_. Each layer of Bi or Bi_2_Se_3_ had a thickness of about 100 nm, and the total length of the nanowire was 10 µm with a diameter of 50 nm (Xue et al., 2014).

Li et al. electrodeposited thick Bi_2_Se_3_ films using ITO-coated glass as substrate in a acidic solution containing 25 mM SeO_2_, 25 mM Bi(NO_3_)_3_ and 1.3 M HNO_3_. The results showed that the as-deposited films were p-type Bi_2_Se_3_ films. The power factors of 52.57 μW/mK^2^ were obtained for the as-deposited films (Xiaolong and Zhen, 2014).

Tumelero et al. (2016) electrodeposited Bi_2_Se_3_ using Si (100) substrate as substrate in the electrolyte consisting of 1.5 mM SeO_2_, 1 mM Bi(NO_3_)_3_ and 0.5 M nitric acid. The results indicated that Bi_2_Se_3_ with single orthorhombic phase and stoichiometric composition can be deposited by tuning the applied potential, while the potential window was narrow. Additionally, the deposited Bi_2_Se_3_ had a bandgap of 1.25 eV (Tumelero et al., 2016).

Souza et al. (2017) synthesized Bi_2_Se_3_ films by potentiostatic electrodeposition method in the electrolyte consisting of 1.5 mM SeO_2_, 0.5 mM Bi_2_O_3_ and 1.0 M HClO_4_ using silicon (100) as substrate. The deposited Bi_2_Se_3_ films is compact with uniform and smooth morphology. The as-deposited films had a dominant orthorhombic phase with mixture of rhombohedral and amorphous phases. However, pure rhombohedral structure was obtained after annealing (Souza et al., 2017).

### Electrodeposition of Bi_2_S_3_ Based Materials

Jagadish et al. (2016) synthesized n-type Bi_2_S_3_ films in the solution consisting of 20.6 mM Bi(NO_3_)_3_, 0.54 M of lactic acid, 0.78 M of nitric acid and 140.8 mM Na_2_SO_4_. Virgin carbon fiber and recycled carbon fiber were used as substrates. The deposited Bi_2_S_3_ had a composition near stoichiometry. The surface morphology and the Seebeck coefficient of Bi_2_S_3_ films can be tuned by post annealing process. The Bi_2_S_3_ films had Seebeck coefficient of -16.3 and -12.4 µV/K deposited on virgin carbon fiber and recycled carbon fiber, respectively (Jagadish et al., 2016).

### Electrodeposition of Sb_2_Te_3_ Based Materials

Ueda et al. (2008) synthesized Sb_2_Te_3_ alloy in the AlCl_3_-NaCl-KCl molten salt electrolyte containing 10 mM TeCl_4_ and 7 mM SbCl_3_, at the temperature of 423 K and applied potential of 0.85 V vs. Al/Al(III). The composition of Sb can be controlled by concentration ratio of the Sb(III) to [Sb(III) + Te(IV)]. The morphology of deposited Sb_2_Te_3_ alloy is disk-like granule, which had a size of around 10 µm (Ueda et al., 2008).

Park et al. (2009) electrodeposited thin Sb_x_Te_y_ films and nanowires at room temperature in an acidic electrolyte. Pt/Si and Au were used as substrate. The applied voltage and film thickness had significant effect on the morphology and grain size of the Sb_x_Te_y_ films. Amorphous Sb_x_Te_y_ films was electrodeposited, while the films became the rhombohedral R3m structure after annealing (Park et al., 2009).

Kim and Oh (2010b) investigated the crystallization behavior of the electrodeposited Sb_2_Te_3_ film in the electrolyte containing 7 mM Te ion, 63 mM Sb ion, 3.5 M perchloric acid, 0.35 M tartaric acid. The transition crystal structure from amorphous to crystalline would influence the Seebeck coefficient. Moreover, the addition of Cu can improve the thermal stability of the Sb_2_Te_3_ film, where CuSbTe film had a crystallization temperature of 149.5°C (Kim and Oh, 2010b).

Pinisetty et al. (2011b) electrodeposited polycrystalline Sb_2_Te_3_ nanowires and nanotubes arrays in the electrolyte consisting of 0.7 mM TeO_2_, 1.6 mM Sb_2_O_3_, 33 or 330 mM tartaric acid, and 3 M HNO_3_. The nanowires and nanotube had an average lamellar thickness of 36 and 43 nm, respectively (Pinisetty et al., 2011b).

Lim et al. (2011) electrodeposited *p*-tyape Sb_x_Te_y_ thin films in an acidic solutions. The effect of TeO_2_ concentrations was investigated. Sb_2_Te_3_ films with composition near stoichiometry was deposited with a rhombohedral structure and preferred orientation of (015). The films had a carrier concentration of 5.8 × 10^18^ cm^−3^ and mobility of 54.8 cm^2^/(Vs). Additionally, more negative applied potential would reduce the carrier concentration and mobility, which is possibly owing to increase in defects. A Seebeck coefficient of 118 μV/K was obtained at room temperature for the as-deposited Sb_2_Te_3_ film (Lim et al., 2011).

Qiu et al. (2011) synthesized Sb_2_Te_x_ (2 < × < 6) films in the alkaline solution with TeO_3_^2-^, SbO_2_^−^, diaminourea polymer and triethanolamine. The solution was pretreated by argon gas to fully deaerate, which would enhance the Seebeck coefficient of the films by reducing oxygen contamination in the deposited films. The Sb_2_Te_x_ films were amorphous before annealing. A maximum power factor 1.58 mW/mK^2^ was achieved with a Seebeck coefficient of 532 μV/K after annealing (Qiu et al., 2011).

Schumacher et al. (2012) electrodeposited Sb_2_Te_3_ films in the electrolyte composing of 10 mM TeO_2_, 5.6 mM Sb_2_O_3_, 0.84 M tartaric acid and 1 M nitric acid with pH of 1. Both Au and stainless steel were used as substrates. The results showed that morphology and composition of the films could be improved by pulsed electrodeposition methods. The p-type Sb_2_Te_3_ films fabricated by pulsed electrodeposition methods achieved a power factors of about 700 μW/(mK^2^) at room temperature with the electrical conductivity of 280 S/cm and Seebeck coefficients of 160 μV/K. Additionally, a maximum power factors obtained is 852 μW/(mK^2^) after annealing (Schumacher et al., 2012).

Li et al. (2012) electrodeposited Sb_x_Te_y_ films in a nonaqueous electrolyte containing 20 mM SbCl_3_, 20 mM TeCl_4_ and 0.1 M KNO_3_. The Sb_x_Te_y_ films had a smooth morphology, which is independent of applied potential. The composition obtained nearest to stoichiometry is Sb_1.87_Te_3.13_. Additionally, all the films were p-type after annealing (Li et al., 2012).

Lim et al. (2012b) synthesized Sb_x_Te_y_ films in the electrolyte with 2.4 mM HTeO_2_^+^, 0.8 mM SbO^+^, 33 mM tartaric acid, and 1 M HNO_3_ by electrodeposition. The thin Sb_2_Te_3_ films with composition near stoichiometry were deposited in the applied potential range of -0.15 to -0.30 V vs. SCE. The post-annealing process would reduce the FWHM of the major diffraction peaks and enhance the electrical conductivity. Additionally, the power factor was improved from 44.2 to 372.1 mW/(mK^2^) by annealing (Lim et al., 2012b).

Lensch-Falk et al. (2012) electrodeposited thin Sb_2_Te_3_ films in the electrolyte consisting of 7 mM sodium tellurite (IV), 16 mM antimony (III) oxide, 0.3 M tartaric acid, and 2 M nitric acid at room temperature by pulsed electrodeposition method. The results showed that the pulse duration have a significant effect on the texture and microstructure of films, where lamellar microstructure was deposited at short pulse durations, while equiaxed and randomly oriented microstructure was deposited at relative long pulse durations. Additionally, reducing pulse duration would also help to suppress the thermal conductivity of the films, where thermal conductivity of less than 2 W/(Km) was obtained (Lensch-Falk et al., 2012).

Nguyen et al. (2013) fabricated Sb, Te and Sb_x_Te_y_ from molten salts containing acetamide - antimony chloride and tellurium chloride by electrodeposition. The Te composition of Sb_x_Te_y_ alloy films is ranged from 20 to 81 at%, which is obtained in the electrolyte with SbCl_3_ up to 0.48 M and TeCl_4_ up to 0.12 M (Nguyen et al., 2013).

**FIGURE 8:**
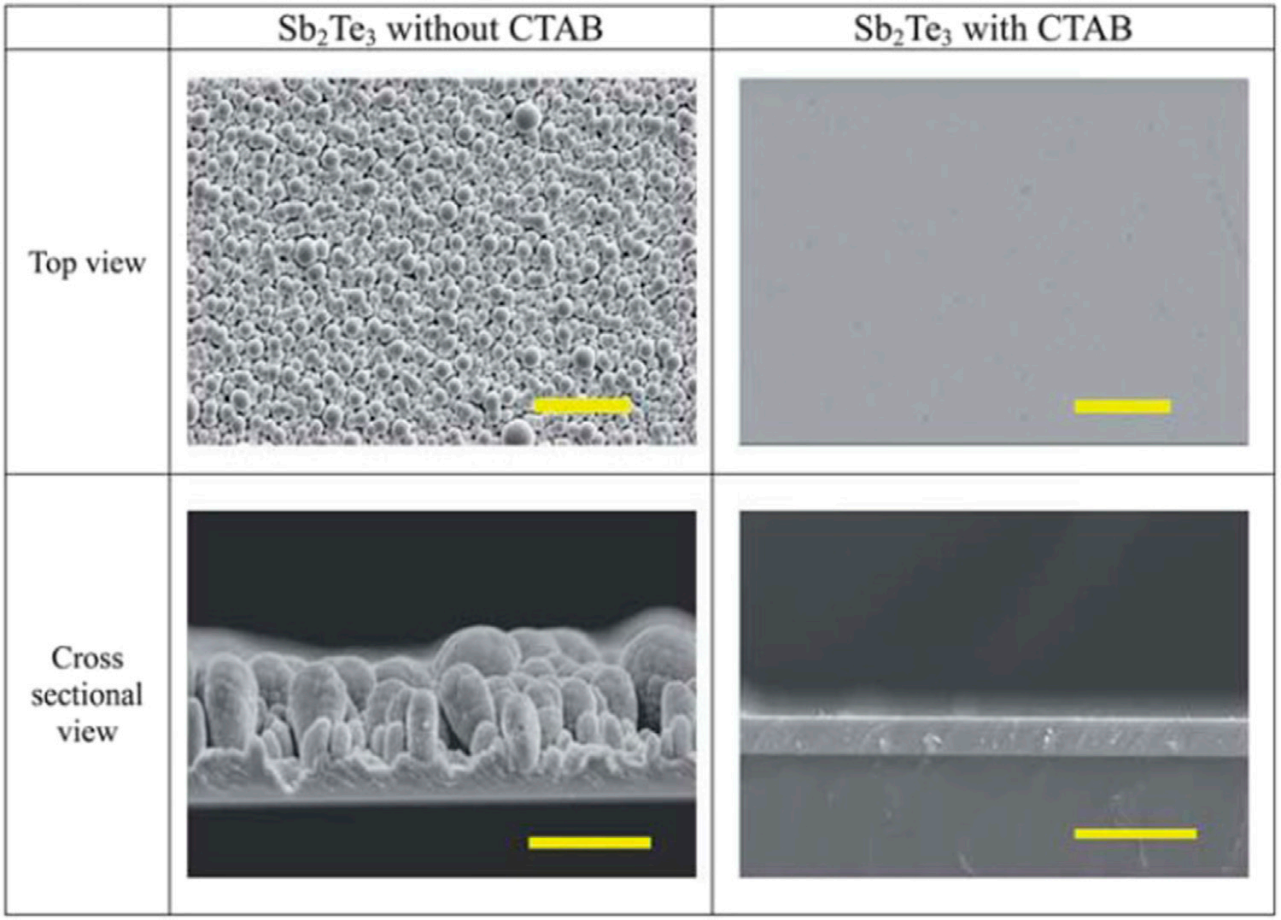
Comparison of the top view (scale bar = 30 µm) and the cross-sectional (scale bar = 20 µm) SEM images of the Sb_2_Te_3_ films electrodeposited with and without CTAB (Yoo et al., 2013c).

Yoo et al. (2013c) synthesized Sb_2_Te_3_ films in the solution consisting of 2.4 mM TeO_2_, 0.8 mM Sb_2_O_3_, 33 mM tartaric acid, and 1 M HNO_3_ at room temperature. Additionally, cetyltrimethylammonium bromide (CTAB) was used as surfactant to improve the surface morphology, where the effect of CTAB on the morphology of Sb_2_Te_3_ films was shown in Figure 8. Moreover, CTAB would also help to enhance the adhesion of Sb_2_Te_3_ films to substrate. Post-annealing at 200°C can improve electrical conductivity and Seebeck coefficient of the Sb_2_Te_3_ films, which was attributed to Te nanodots formation within the Sb_2_Te_3_ crystal structure. A power factor of 716.0 mW/mK^2^ was obtained for Sb_2_Te_3_ films with 10–20 nm Te nanodots (Yoo et al., 2013c).

Kim et al. (2015) synthesized Te-rich Sb_2_Te_3_ film in a solution with 3.6 mM Sb_2_O_3_, 2.4 mM TeO_2_, 33 mM L-tartaric acid, 1 M HNO_3_. The as deposited films were amorphous, while γ-SbTe embedded nanocrystalline Sb_2_Te_3_ film was obtained by post annealing process because of solid-state phase transition. The results indicated that γ-SbTe embedded Sb_2_Te_3_ had higher Seebeck coefficient and P.F. than single phase Sb_2_Te_3_ film. This was attributed to strong energy-dependent charge scattering, which is confirmed by UPS analysis showing 90 meV valence band difference between Sb_2_Te_3_ and γ-SbTe nanocrystalline. As a consequence, a Seebeck coefficient of 320 μV/K was obtained for γ-SbTe/Sb_2_Te_3_ nanocomposite (Kim et al., 2015).

**FIGURE 9:**
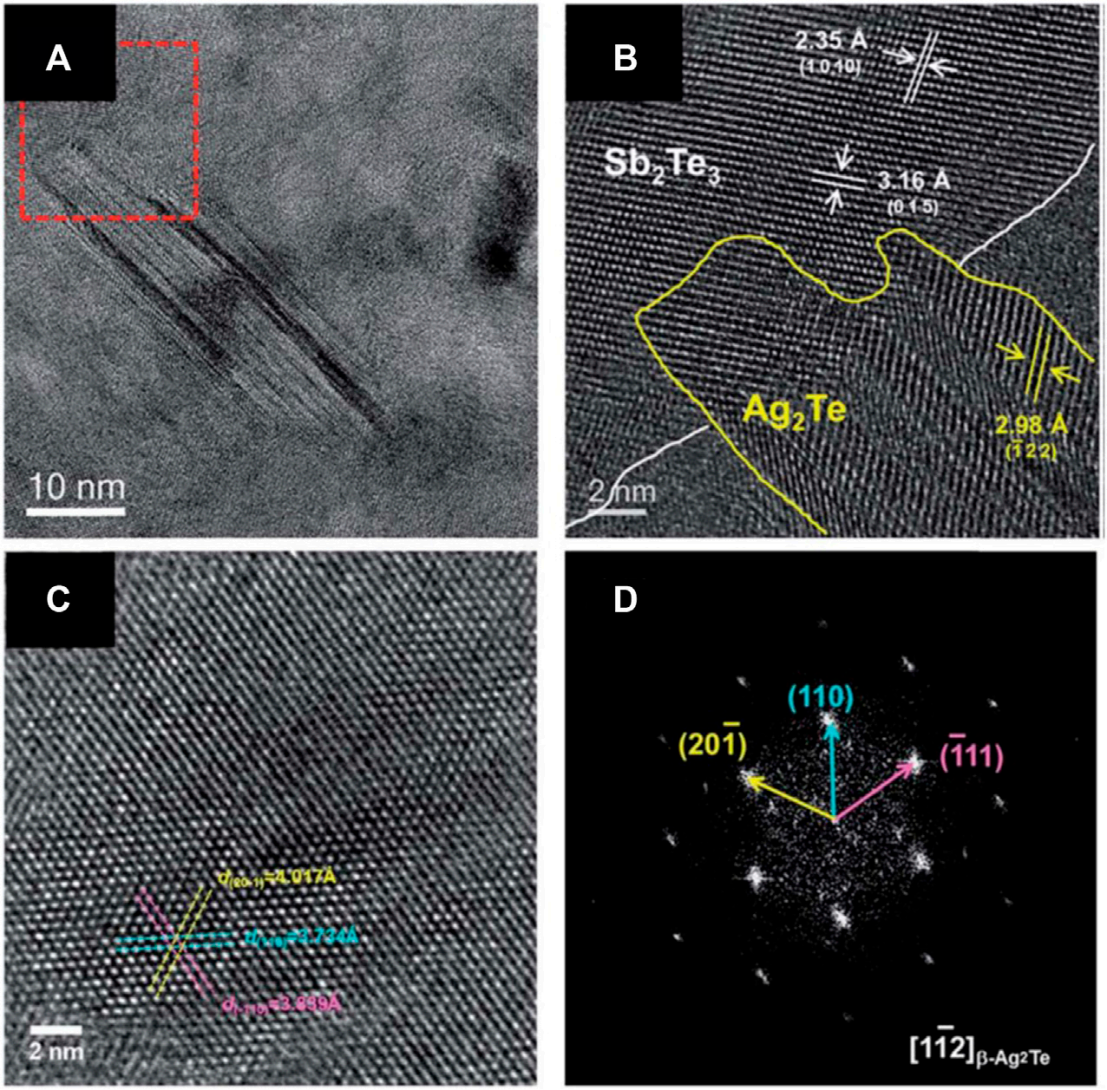
Microstructure of the precipitated Ag2Te phase embedded in Sb2Te3 film. **(A)** HRTEM image and **(B)** a lattice image showing the precipitated Ag2Te nanodots within the Sb_2_Te_3_ matrix. **(C)** HRTEM image taken at a (Nolas et al., 2001; Snyder and Ursell, 2003; Hsu et al., 2004; Zide et al., 2006; Zeng et al., 2007; Faleev and Léonard, 2008; Poudel et al., 2008; Snyder and Toberer, 2008; Sootsman et al., 2009; Ko et al., 2011; Szczech et al., 2011; Zebarjadi et al., 2012) zone axis and **(D)** the corresponding FFT image (Kim et al., 2018b).

Kim et al. (2018b) also electrodeposited Ag_2_Te nanoprecipitates embedded p-type Sb_2_Te_3_ films (Figure 9). The same electrolyte condition and applied potential (0.1 vs. SCE) was applied to deposit the films except adding 100 μM AgNO_3_ as Ag sources. The results indicated that the presence of the β-Ag_2_Te phase would improve the Electrical property Sb_2_Te_3_ films dramatically, which was attributed to energy-dependent charge carrier filtering effect at the β-Ag_2_Te/Sb_2_Te_3_ interface. Additionally, density of states effective mass (m*∼1.8 m_0_) increased, leading to a high power factor of 1870 mW/mK^2^ at 300 K without any dramatic suppression of electrical conductivity (Kim et al., 2018b).

Catrangiu et al. (2016) electrodeposited Sb_2_Te_3_ film in the ionic liquid with 4–10 mM TeO_2_, 4–10 mM SbCl_3_, choline chloride and oxalic acid. The composition of the films can be controlled by precursor concentration and applied potential. The electrodeposited mechanism is that Te layer was deposited followed by the deposition of Sb_x_Te_y_ compounds at more negative applied potential (Catrangiu et al., 2016).

Hatsuta et al. (2016) synthesized Sb_2_Te_3_ thin films in the solution consisting of 1.6 mM TeO_2_, 1.3 mM SbF_3_, and 0.39 M HCl by electrodeposition using stainless steel as substrate. The results indicated that a stoichiometric atomic composition was achieved for the as-deposited thin film. Moreover, the composition of thin film kept at stoichiometry after annealed at the temperature below 250°C. However, when the annealing temperature go up to 300°C, a portion a of alien element, including Fe, Cr, Ni, was detected in the film, which lead to lower Seebeck coefficient and higher electrical conductivity. As a consequence, a maximum power factor of 13.6 µW/(cmK^2^) was obtained for the Sb_2_Te_3_ film (Hatsuta et al., 2016).

Kim et al. (2016) electrodeposited thin AgSbTe_2_ films. The deposited amorphous Ag_15_Sb_27_Te_58_ film showed a Seebeck coefficient of 1,270 µV/K. The carrier concentration of about 10^16^ to 10^19^ cm^−3^ was obtained. For deposited nanocrystalline film, The power factors of 90–553 mW/mK^2^ was obtained owing to higher Hall mobility and Seebeck coefficients (Kim et al., 2016).

## Conclusion

Electrochemical deposition is a cost effective and manufacturable synthesis method, which can be used to deposit thermoelectric materials with controlled morphology, composition and crystal structures. The electrodeposition baths including aqueous solution (*e.g.,* acidic and alkaline solutions), ionic liquid, deep eutectic solvents solutions were used to deposit TE materials. Most of the papers investigated the electrodeposition mechanism and kinetics, and the control of morphology, composition and crystal structure of the deposits by electrodeposition parameters, such as precursor concentration, solution pH in aqueous solution, agitation, additives, temperature, applied potential/current, substrate and so on. The correlation between electrodeposition parameters and TE properties was reported, which is indirect correlation because the material properties (*e.g.,* morphology, composition and crystal structure) are the direct effects on the TE properties. The correlation between material properties and TE properties was also discussed by various groups.

Thermoelectric micro-devices have a great potential to serve as a generator/cooler for portable electronic devices. Electrodeposition have an advantage to be utilized to fabricate TE micro-devices, attributed to its ability to deposit thick films, which can be used to fabricate cross-plane devices, with controlled morphology, composition, and crystal structure. The performance of the TE micro-devices are for now limited because of low efficiency. More researches about thermoelectric properties of electrodeposited materials and the performance of devices need to be further studied for wide applications.
